# Bench testing a contact sensing tracheal tube for monitoring the cuff–trachea interface

**DOI:** 10.1186/s42490-026-00113-y

**Published:** 2026-06-01

**Authors:** Tamaralayefa B. Agbiki, Sandor Erdody, Ricardo Correia, Sergiy Korposh, David W. Hewson, Andrew M. Norris, Barrie R. Hayes-Gill, Stephen P. Morgan

**Affiliations:** 1 Optics and Photonics Research Group, Faculty of Engineering, University of Nottingham, Nottingham, UK; 2 Medical Photonics Ltd, Nottingham, UK; 3 Anaesthesia and Critical Care, Academic Unit of Injury, Recovery and Inflammation Sciences, School of Medicine, University of Nottingham, Nottingham, UK

**Keywords:** Tracheal intubation, Cuff monitoring, Optical fibres, Sensors, Tracheal seal testing

## Abstract

Cuffed tracheal tubes enable a secure airway during mechanical ventilation; however, improper cuff inflation can lead to complications such as inspiratory gas leaks, pulmonary ingress of orogastric secretions, or tracheal injury. This study explores a sensor-based approach to optimise cuff inflation using fibre Bragg grating (FBG) optical sensors embedded within the cuff to detect contact with the tracheal wall. Testing was conducted on multiple trachea models, including cylindrical and bio-inspired models, as well as ex vivo porcine trachea samples, to assess cuff–trachea contact. Seal performance was evaluated at both standard inflation pressures and contact-guided inflation pressures. The contact-sensing tracheal tube successfully detected cuff–trachea contact in all models and ex vivo samples tested. Seal tests showed that larger diameter tracheal models exhibited lower leakage rates at contact-guided inflation pressures compared with standard inflation, indicating improved sealing. In contrast, smaller diameter models showed higher leakage rates at contact-guided pressures due to cuff folding, which formed leakage pathways. These results demonstrate that contact sensing improved cuff performance in larger tracheal models by enhancing sealing effectiveness. The contact-sensing tracheal tube differentiated tracheal model sizes, indicating potential for personalised cuff inflation to accommodate variations in tracheal size and shape. Future work will focus on further sensor miniaturisation and integration within lower-volume cuffs to enable effective contact sensing across a wider range of tracheal geometries and support translation to in vivo use.

**Supplementary Information:**

The online version contains supplementary material available at 10.1186/s42490-026-00113-y.

## Introduction

Tracheal intubation is a common and vital procedure in many patients who require mechanical ventilation while sedated during critical illness or anaesthetised for surgery. In the UK alone, over one million intubations are performed annually [1]. The tracheal tube provides a reliable conduit for positive pressure ventilation of the lungs, and the inflatable cuff aims to create a gas-tight seal and to prevent contamination of the lungs by gastric secretions or other material [2].

Optimal tracheal tube cuff pressure is important. If pressure is too low, the cuff will not adequately contact the trachea wall, resulting in (i) poor gas seal, increasing the risk of ventilation failure and hypoxia, and (ii) leakage of secretions from the oropharynx to the lungs, placing the patient at risk of Ventilator Associated Pneumonia (VAP) [3, 4]. If cuff pressure is too high, the cuff restricts blood flow in the tracheal mucosa leading to ischaemia-related inflammation and tissue necrosis, ulceration, scarring and laryngo-tracheal stenosis as well as other pressure related complications [4–10]. For lengthy intubations (common in critically ill patients in intensive care), compromised trachea mucosal perfusion causes chronic inflammation, tracheal necrosis and airway stenosis. These health consequences highlight a need for technological solutions to maintain personalised tracheal tube cuff pressure optimised for the patient’s anatomical and physiological requirements during the period of intubation.

Intra-cuff inflation pressure is often used as a surrogate for cuff–trachea contact pressure, however, this can differ significantly between patients as it does not account for cuff shape, or volume variations. This highlights the need for direct monitoring of cuff contact to ensure an appropriate seal.

Previous innovations in anaesthesia have incorporated pressure monitoring to assist with the intubation process but none have used sensors embedded in the tracheal tube cuff to inform cuff contact. The Venner PneuX uses an external trachea seal monitor to maintain the intracuff pressure at a selected value [11]. The TRACOE smart Cuff Manager is an external device used to maintain cuff pressure within a specified range [12]. Goethals et al. attached an elastoresistive sensor to the outer wall of the cuff to detect the presence or absence of tracheal rings for tracheal tube placement, using a simplified rigid tracheal model with discrete ring structures [13]. While this demonstrated the feasibility of cuff-integrated sensing, the approach was limited to positional detection and did not directly inform cuff–trachea contact. More broadly, sensing-enabled airway devices have been explored to provide physiological feedback during airway management. For example, smart airway stents incorporating integrated sensors have been developed to monitor local mechanical and biological conditions, with increasing interest in bioabsorbable and multifunctional platforms [14]. In addition, sensing systems for respiratory monitoring have been widely investigated, covering sensing modalities including pressure, optical, and impedance approaches for the measurement of respiratory rate and airflow [15]. Our research group has developed an intra-tracheal multiplexed sensing (iTraXS) tracheal tube, deploying optical fibre sensors to measure cuff contact pressure and mucosal perfusion [16]. Building on this concept, the present work explores using sensors embedded in a single cuff to indicate contact between the cuff and trachea in a model system.

The current clinical standard of care is a ‘one-size-fits-all’ maximum inflation pressure of 30 cmH_2_O for all adult patients (Royal College of Anaesthetists, American Society of Anaesthesiologists, European Task Force on ventilator-associated pneumonia) [17, 18]. A tracheal tube which could inform adequate cuff pressure by indicating when contact has been made with the trachea wall would support achieving a better seal without over-inflation of the cuff. This has the potential to support clinicians and provide a personalised approach to improve patient outcomes.

The aim of this study was to investigate whether a tracheal tube with a contact sensor-equipped cuff, used to inform inflation pressure, can provide a safe tracheal seal in a benchtop model system. The main objectives were (1) fabricate a contact-sensing tracheal tube using Fibre Bragg Grating (FBG) sensors; (2) investigate the relationship between inflation pressure and cuff–trachea contact for a range of cylindrical trachea models, a bio-inspired trachea model and ex vivo porcine trachea; (3) conduct tracheal seal tests (ISO 5361:2023) to compare model-specific contact sensor guided (contact-guided) inflation pressure with the ‘one size fits all’ (standard) inflation pressure.

## Methods

### Contact-sensing tracheal tube fabrication

There were two stages in the fabrication process of the contact-sensing tracheal tube. First, fabrication of the FBG cantilever contact sensor. Second, insertion of the sensor into the tracheal tube cuff.

**Fig. 1:**
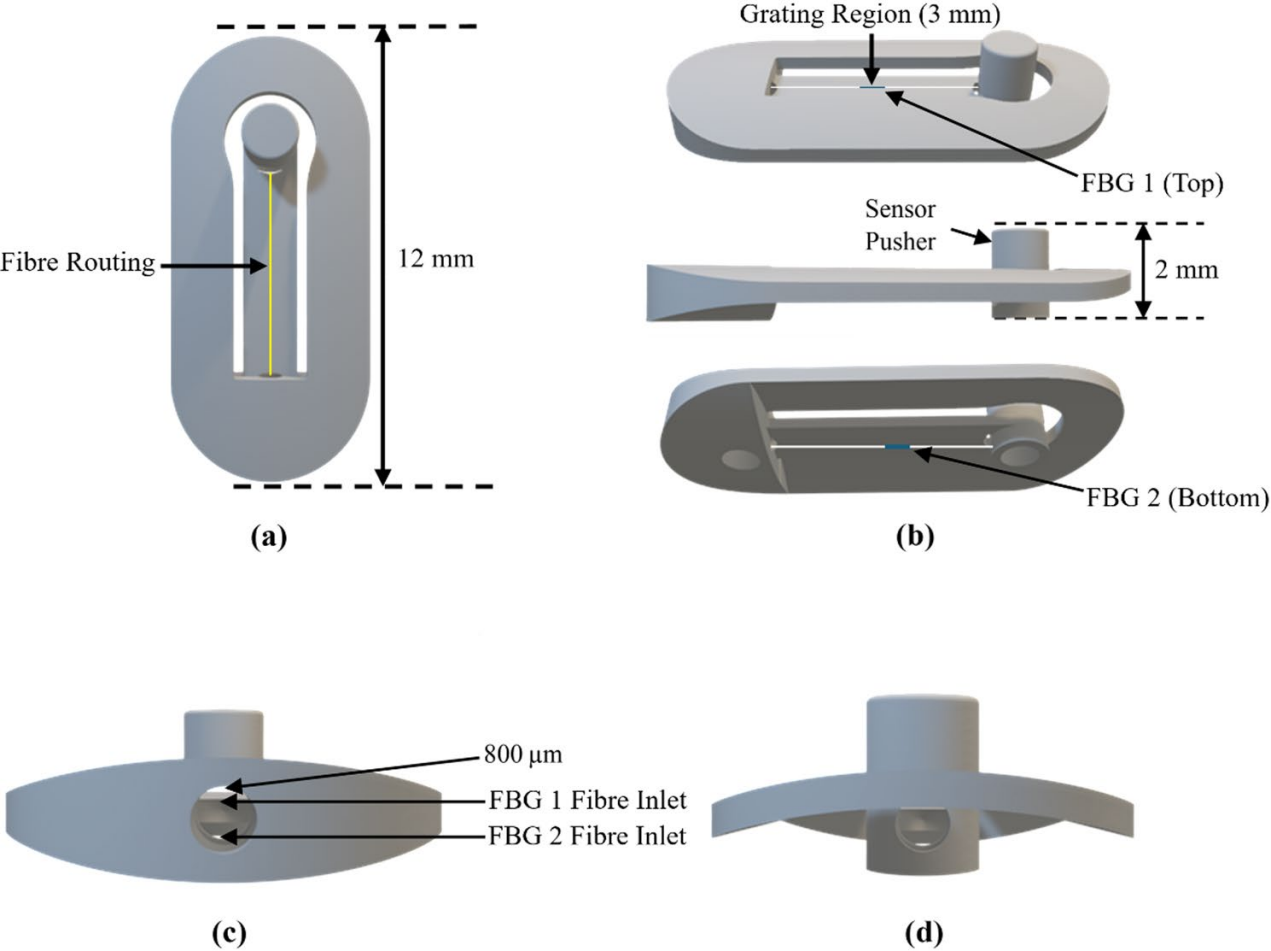
3D-printed cantilever design for FBG contact sensor, showing the cantilever structure and fibre routing. **a** Front view; **b** side view; **c** bottom view; **d** top view

A 3D design of the cantilever sensor is shown in Fig. 1.

The sensor uses two fibre Bragg gratings inscribed on single-mode optical fibres (core/cladding diameter 8.2/125 µm) with central wavelengths of 1540 nm and 1550 nm (acrylate-coated SMF-28 from Corning, FBGs inscribed by Samyon Instruments, CN), each with a sensing length of 3 mm, attached to a 3D-printed holder incorporating a cantilever that responds to applied pressure. The optical fibres were inserted through the base of the 3D-printed sensor holder and bonded across the grating region on the upper and lower surfaces of the cantilever using a UV-cured medical adhesive (Loctite^®^ AA 3926, Henkel, DE) to form a differential sensing configuration, before exiting the rear of the holder. The configuration of the cantilever sensor, including the FBG locations, fibre routing, and key dimensions, is illustrated in Fig. 1.

The sensor holder was designed and 3D printed (Form 3B+, FormLabs, US) using biocompatible resin. A single FBG responds to strain on the fibre exerted by pressure applied to the cantilever; however, temperature variations also influence the Bragg wavelength. The use of two FBGs on opposite cantilever surfaces enables differential measurement, which enhances sensitivity and compensates for temperature cross-sensitivity. A 50 g mass was suspended at the grating end of the fibres to pre-strain them prior to curing, which enhances the sensitivity of the FBGs [19]. After curing, the fibres were inserted into a fibre jacket (900 μm furcation tubing, FT900Y, Thorlabs, UK) for protection from direct mechanical forces.

The cantilever incorporates a pusher which causes the cantilever to bend when pressure is applied. Cantilever deflection produces tensile strain in the upper FBG and compressive strain in the lower FBG, resulting in opposite wavelength shifts. Subtracting the wavelength shift of the lower FBG from the upper FBG enhances sensitivity and cancels the temperature response common to both sensors [20]. An illustration of the differential FBG response to applied pressure is shown in Fig. S1. The differential wavelength shift, demonstrating stable and repeatable pressure sensitivity during calibration against a digital manometer (Extech HD750, Teledyne FLIR, US), is shown in Fig. S2.

**Fig. 2:**
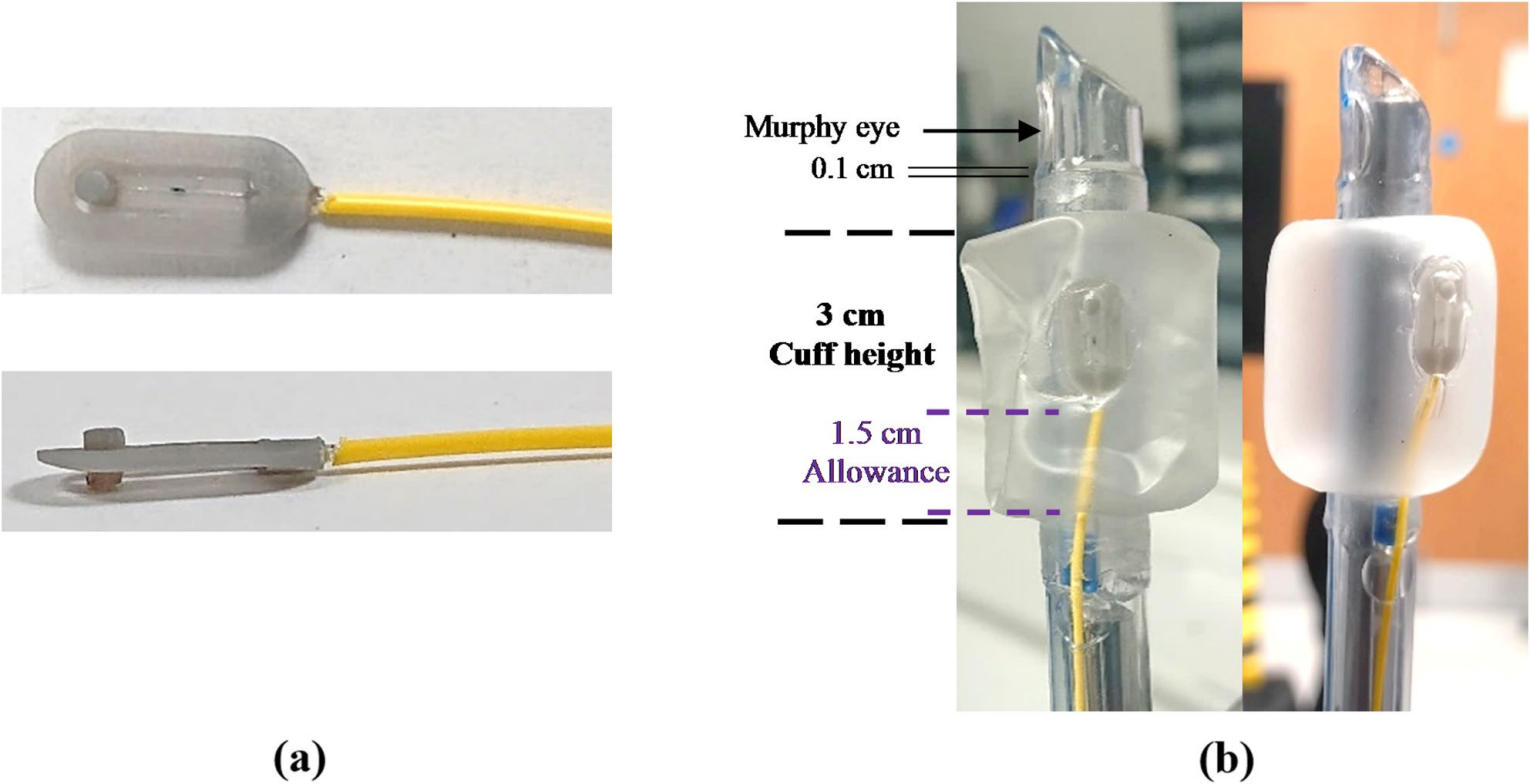
Fabrication of contact-sensing tracheal tube for contact monitoring. **a** Contact sensor with 2 FBGs inserted; **b** finished version of contact-sensing tracheal tube; deflated and inflated

The second fabrication stage involved inserting the sensor (fabricated version shown in Fig. 2a) into the tracheal tube cuff, with the final contact-sensing tracheal tube shown in Fig. 2b.

A size 8 mm lumen (outer diameter (OD): 10.9 mm) was used for tracheal tube fabrication (P3 Medical, UK). The optical fibres were passed through the subglottic suction lumen of a tracheal tube to provide a means of monitoring the sensor from the proximal end. The proximal ends of the fibres were then spliced to a fibre optic pigtail containing LC/APC connectors (FP9LCAPC, all4fibre, AT) using a fusion splicer (FSM-100P, Fujikura, JP) to enable connection to the interrogation unit (Sentea DM-8125, Sentea, BE). After splicing, the sensor holder (including the pusher) was attached to the inner surface of a high-volume low-pressure (HVLP) size 8 (Ø 31) cuff using UV cured adhesive.

The bottom end of the cuff was slightly stretched, and the cuff was placed 0.1 cm above the Murphy eye on the tracheal tube lumen. The sensor was adjusted to prevent kinks in the cuff when inflated and a 1.5 cm inflation allowance was applied to the fibre to prevent additional strain before sealing the top and bottom end of the cuff with UV cured adhesive. After fabrication, the tube was tested by inflation to confirm that there were no air leaks using a syringe, 3-way stopcock and digital manometer.

### Tests to investigate the relationship between the cuff and trachea model wall contact and inflation pressure

**Fig. 3:**
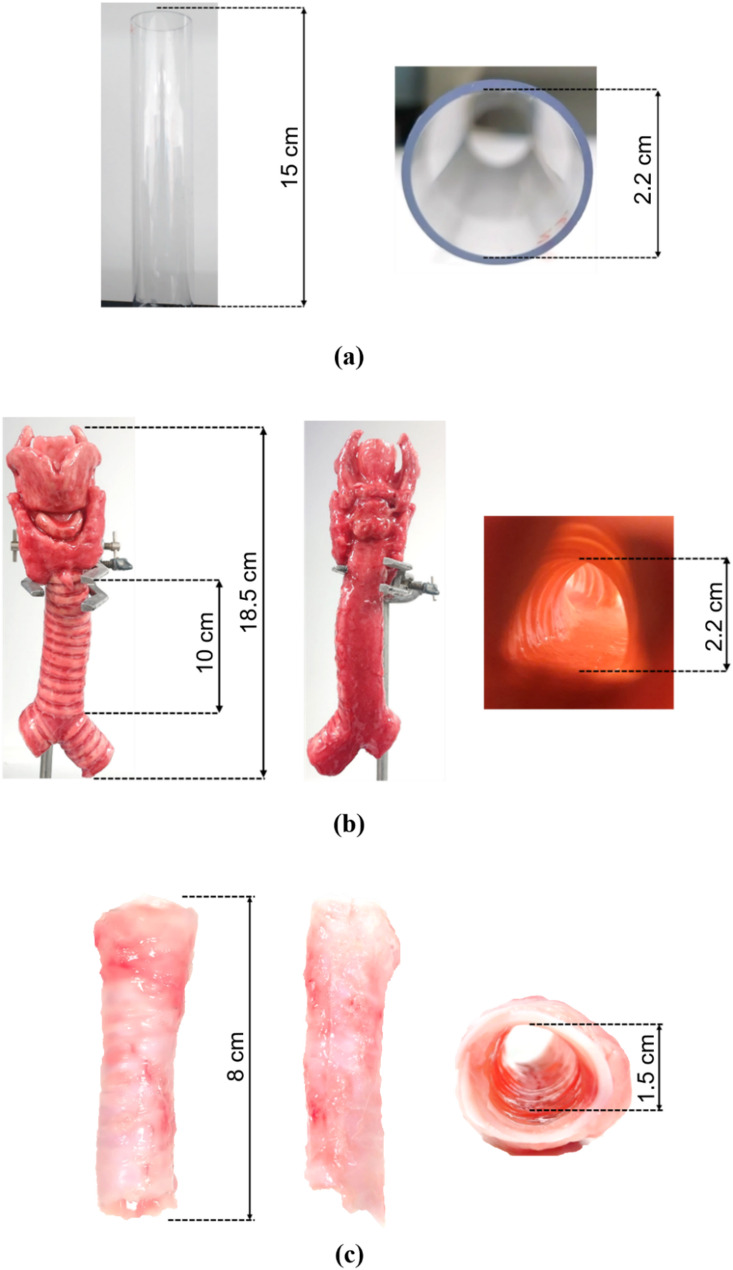
Trachea models. **a** Cylindrical model (as per the ISO 5361:2023 standard), showing external and internal views; **b** bio-inspired model, showing external views of the anterior and posterior aspects, and internal view of the tracheal lumen; **c** ex vivo porcine trachea, showing external views of the anterior and posterior aspects, and internal view of the tracheal lumen

Cuff contact tests were conducted using five cylindrical trachea models of varying diameters (representative example shown in Fig. 3a), a bio-inspired trachea model [21] (Fig. 3b) and three ex vivo porcine trachea samples (representative example shown in Fig. 3c) to find the inflation pressure at which the cuff contacts the wall of each model.

The bio-inspired model was fabricated using a computerised tomography (CT) scan of a non-pathological adult human trachea, replicating its anatomical geometry and mechanical properties [21]. The model incorporates a stiff, C-shaped cartilage ring structure and a softer, continuous posterior wall representing the trachealis muscle, thereby capturing the dominant mechanical heterogeneity of the human trachea. The phantom was fabricated using PDMS gels (Platsil^®^ Gel 00–30 and Transil^®^ 20) modified to represent different tracheal regions, with the mucosal surrogate exhibiting a Shore hardness of approximately 35–40 Sh00 (corresponding to reported Young’s modulus values of 4–18 kPa) and the cartilage components ~ 60 ShA, within the reported range for tracheal cartilage (59.6–91 ShA; 3.2–23 MPa). This configuration provides a mechanically relevant platform for assessing cuff–trachea contact under controlled benchtop conditions.

Three ex vivo porcine tracheas, sourced postmortem from adult female Tamworth pigs (70 ± 2 kg), were used for testing. Porcine tracheas were selected due to their anatomical and mechanical similarity to the human trachea [22, 23]. The samples were stored and transported in sealed bags on wet ice to preserve tissue integrity prior to testing.

**Fig. 4:**
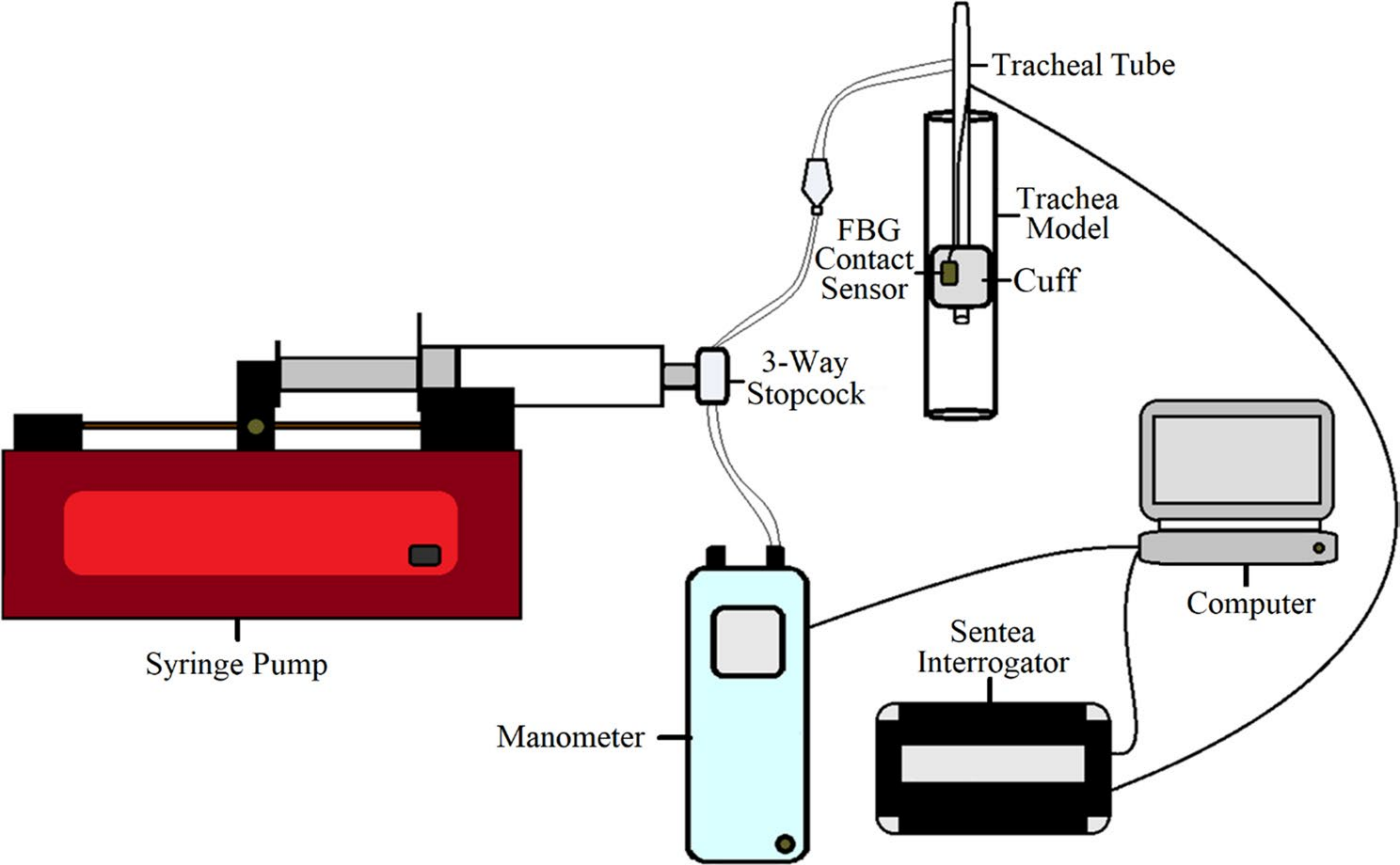
Experimental set-up for cuff–trachea contact test

A block diagram of the cuff–trachea contact test experimental set-up is shown in Fig. 4.

The tracheal tube was connected using a 3-way stopcock linking the pilot balloon of the tube to a programmable syringe pump (Aladdin AL-1000, WPI, UK) and digital manometer, for cuff inflation and to measure the intracuff pressure respectively. The optical fibres were connected to an interrogator unit for data acquisition and signal processing. Intracuff pressure was obtained simultaneously using the digital manometer.

The contact test involved inflation and deflation cycles. Starting with a 1-minute baseline at 0 cmH_2_O, the cuff was linearly inflated to 100 cmH_2_O over approximately 30 s, maintained for 1 min, and then deflated back to 0 cmH_2_O over 30 s, followed by a 1-minute hold at 0 cmH_2_O. The maximum inflation pressure was ~ 100 cmH_2_O to determine whether there are any notable variations in the contact signal at higher pressures. The contact test was performed on five cylindrical tracheal models (internal diameter (ID) 15 mm, 20 mm, 22 mm, 24 mm and 26 mm), one bio-inspired trachea model (ID 22 mm), and three ex vivo porcine trachea samples. Each measurement was repeated six times for each cylindrical model, and for the bio-inspired model on both cartilage and muscle regions (six repeats per region). For the ex vivo samples, four repeats were performed per region due to tissue degradation during repeated testing. Repeats were conducted by removing and reintroducing the tracheal tube into the model. The approximate duration for each test was 4 min, with a total test time of approximately 6 h conducted across two measurement sessions. The contact point was defined as the onset of reversal in the FBG signal during cuff inflation, identified as the first change in signal trend direction. A minimum threshold of 0.01 nm was applied to ensure that detected changes exceeded the system noise level (peak-to-peak baseline variation ~ 0.0024 nm). In cases where a reversal was not clearly distinguishable, the contact point was identified as the first local plateau during inflation, defined as a region where the signal variation remained within ± 0.005 nm for at least 0.5 s (approximately 50 consecutive data points). The inflation pressure at contact was calculated as the mean manometer pressure over the first three consecutive data points following this point.

### Tracheal model seal tests

**Fig. 5:**
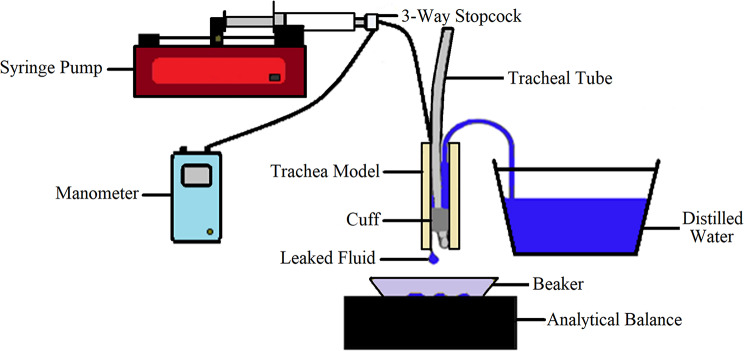
Experimental set-up for tracheal seal test (system diagram adapted from EN ISO 5361:2023)

The leakage of fluid past the cuff of the contact-sensing tracheal tube was investigated. A block diagram of the tracheal seal test set-up adopted from Annex F of EN ISO 5361:2023 is shown in Fig. 5.

The tracheal tube was inserted into the model, and the cuff was inflated to the required pressure via a 3-way stopcock linking the pilot balloon to a programmable syringe pump and digital manometer. Distilled water was then poured into the model and maintained to a height of 5 cm above the cuff. A beaker was placed on an analytical balance (MC1 Laboratory LC4800-P00V, 30906009, Sartorius, DE) to collect leaked fluid beneath the rig. After 10 min, the amount of leaked fluid was recorded. The tracheal tube cuff inflation pressure was set and maintained at a constant value during each experiment. Experiments were performed in ambient conditions, and the temperature of the distilled water was maintained at 37 °C using a water bath (SUB Aqua 5, Grant Instruments, UK). The tracheal tube and trachea models were conditioned for 20 min in the water bath at 37 °C, then air-dried before commencing the experiments. The tracheal tube cuff was inflated and inspected for mechanical integrity before each test.

It is not feasible to conduct measurements of fluid leakage past the cuff in vivo and therefore in vitro models of the trachea were required. Seal tests were conducted using the same five cylindrical trachea models and the bio-inspired trachea model. Two sets of seal tests were carried out for each trachea model to compare the ‘one-size-fits-all’ inflation pressure with the contact sensor guided inflation pressure. For the first set of tests, the cuff pressure was maintained at 27 cmH_2_O (as per EN ISO 5361:2023). For the second set of tests, the pressure was maintained at the contact-guided pressure established for the trachea model being tested. Experiments took a total of 12 h and were carried out in two measurement sessions spread equally over two days.

### Statistical analysis

Statistical comparisons across contact and leakage test results were conducted using a paired *t*-test or the Wilcoxon signed-rank test, depending on normality (assessed using the Shapiro–Wilk test), as measurements were obtained either as paired observations from the same experimental models under different test conditions or from matched models of the same internal diameter. Statistical significance was set at *p* < 0.05.

## Results

### Tests to investigate the relationship between the cuff and trachea model wall contact and inflation pressure

**Fig. 6:**
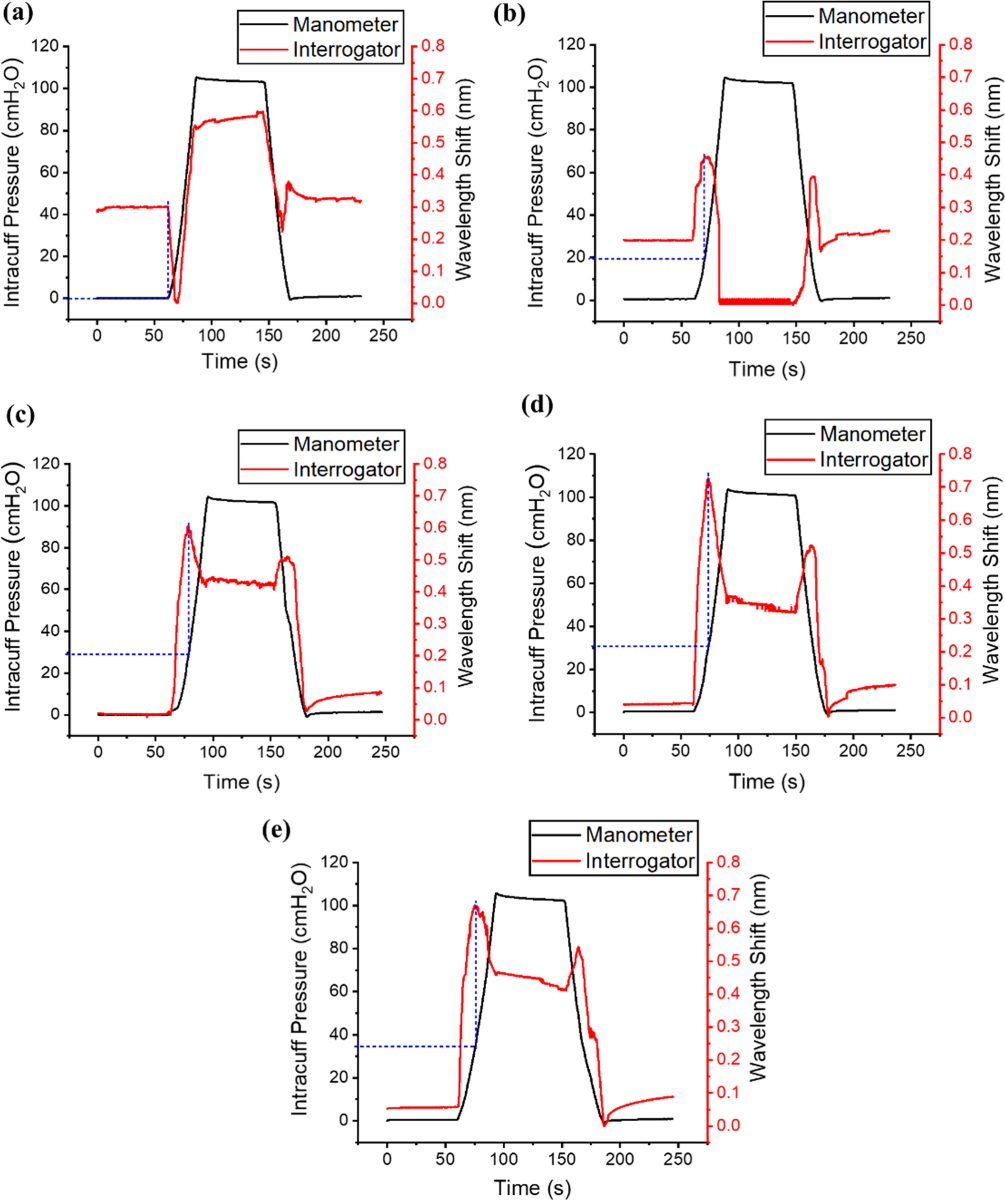
Contact test results for cylindrical trachea models. The blue dashed line indicates the inflation pressure at which contact occurs. **a** 15 mm ID; **b** 20 mm ID; **c** 22 mm ID; **d** 24 mm ID; **e** 26 mm ID

Contact sensor (differential wavelength shift) and manometer data for the five cylindrical trachea models are shown in Fig. 6. The blue dashed line indicates the contact point, corresponding to the inflation pressure at which contact is identified.

Contact test data for the 15 mm ID cylindrical model are shown in Fig. 6a. Pressure was maintained at 0 cmH_2_O for the first minute before inflation. At inflation, the FBG signal moved in the opposite direction to the manometer signal from the onset of inflation, indicating that the cuff was already in contact with the tracheal wall. The signal continued in this direction until the maximum limit of the sensor pusher, after which it reversed and increased in the direction of the inflation pressure until the maximum inflation pressure (100 cmH_2_O) was reached. The signal then remained constant while the cuff inflation pressure was maintained. Deflating the cuff, the FBG signal moved in the same direction as the manometer signal, moving downwards with decreasing cuff pressure. This continued until the sensor pusher returned to the pusher operation range, at this point the FBG signal reversed and proceeded to increase, moving in the opposite direction to the manometer signal until the end of the deflation cycle.

The contact test responses for the 20 mm ID, 22 mm ID, 24 mm ID and 26 mm ID cylindrical models are shown in Fig. 6b, c, d and e respectively. These responses are different to the 15 mm ID model, but all follow the same trend in the inflation and deflation cycles. Pressure was maintained at 0 cmH_2_O for the first minute before inflation. At inflation, the output signal from the FBG increased with inflation pressure, the interrogator and manometer signals moved in the same direction. Both signals continued with this trend until the FBG sensor pusher contacted the trachea model. At contact, the onset of reversal in the FBG signal relative to the inflation pressure indicates the contact point, after which the signal continued until the maximum inflation pressure was reached, and both signals remained constant while pressure was maintained. The deflation cycle followed a reverse trend to the inflation cycle. The FBG signal increased in the initial period of deflation, moving in the opposite direction to the manometer signal until the sensor was no longer in contact with the trachea model. When no longer in contact, the FBG signal changed direction and resumed following the manometer signal trend until the end of the deflation cycle.

**Fig. 7:**
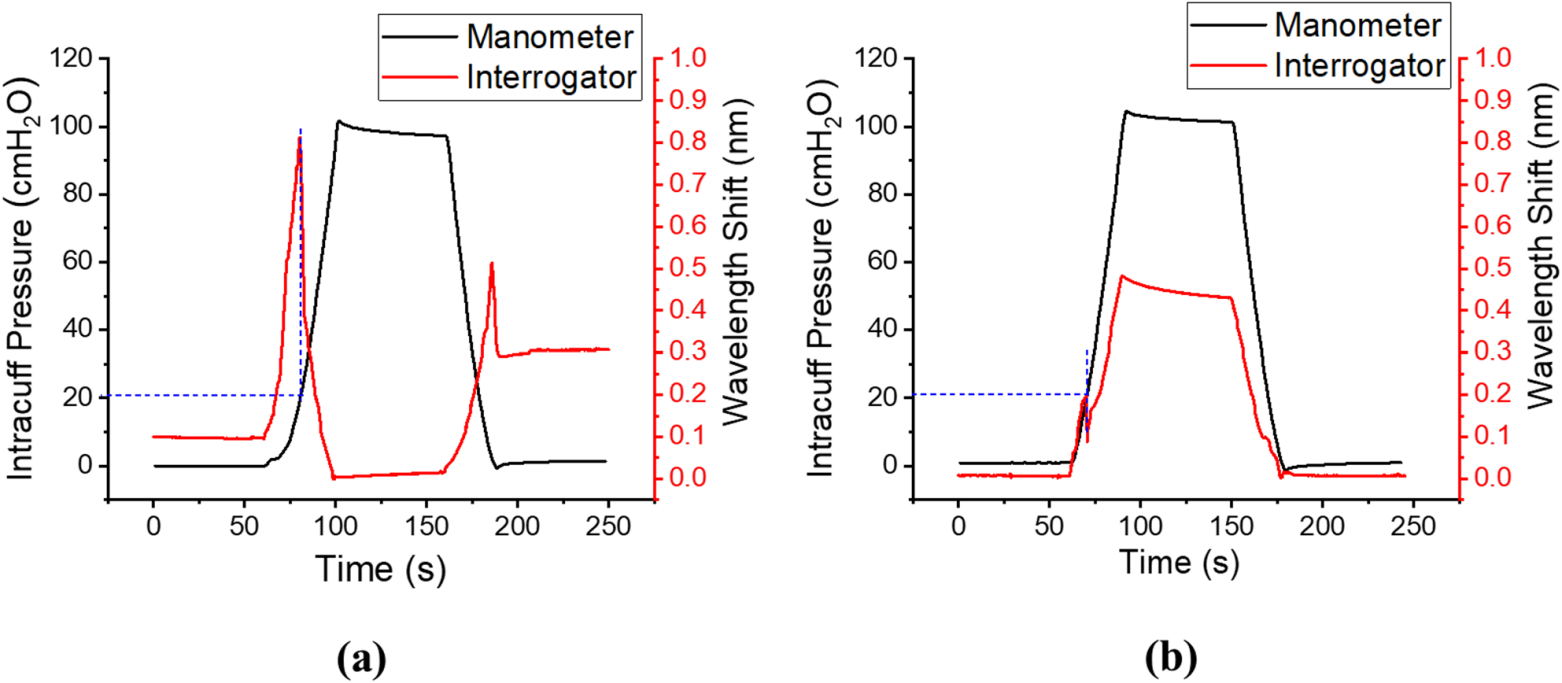
Contact test results for 22 mm ID bio-inspired trachea model. The blue dashed line indicates the inflation pressure at which contact occurs. **a** Cartilage; **b** muscle

Contact tests for the bio-inspired model were conducted with the sensor placed on the cartilaginous aspect (anatomically anterior) first and then on the muscular aspect (anatomically posterior) of the model. Contact test responses for the cartilage and muscle are shown in Fig. 7a and b respectively.

The cartilage contact test response (Fig. 7a) follows a similar trend to the larger sized cylindrical models. During inflation, the FBG signal followed the manometer signal trend increasing until the cuff contacted the trachea model. At contact, the onset of reversal in the FBG signal relative to the inflation pressure indicates the contact point, after which the signal continued until the maximum inflation pressure was reached. The FBG signal remained constant while the cuff inflation pressure was maintained at a constant value until the deflation cycle. During deflation, the FBG signal moved in the opposite direction to the deflation pressure until there was no longer contact. The FBG signal then resumed following the manometer signal trend until the end of the deflation cycle.

The response for the bio-inspired model muscle contact test (Fig. 7b) has a different shape from the cartilage test. During inflation, the FBG signal followed the manometer signal direction increasing until contact, consistent with the cartilage result. At contact, the FBG signal briefly reversed direction relative to the inflation pressure, before subsequently increasing again with the manometer signal until the maximum inflation pressure was reached. For the deflation cycle, the FBG signal decreased as the pressure was reduced, moving in the same direction as the manometer signal. When there was no more contact, the gradient of the FBG signal changed and continued in the direction of the manometer until the end of deflation cycle.

**Fig. 8:**
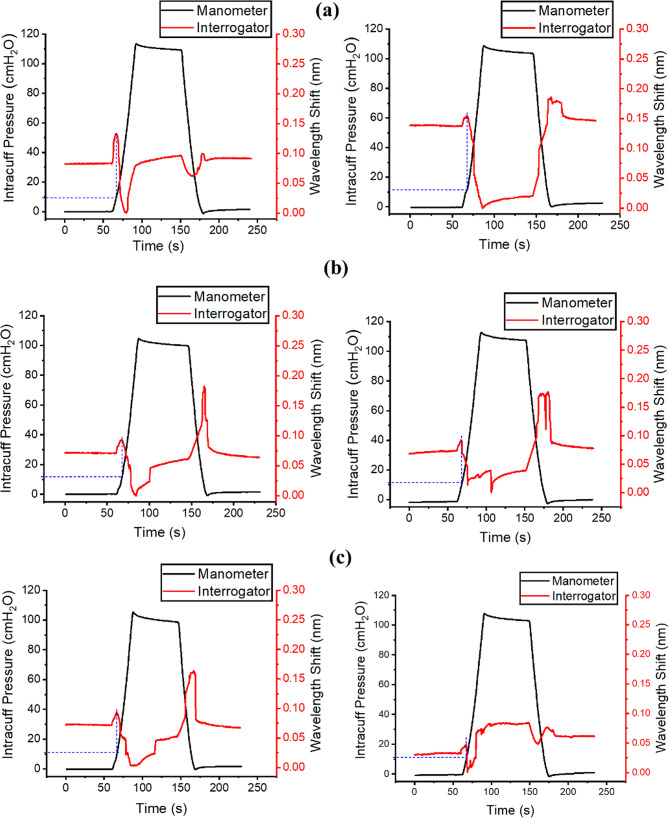
Contact test results for 15 mm ID ex vivo porcine trachea samples, cartilage (first column) and muscle (second column). The blue dashed line indicates the inflation pressure at which contact occurs. **a** Sample 1; **b** sample 2; **c** sample 3

Contact tests for the ex vivo porcine trachea were conducted with the sensor placed on the cartilaginous side first and then on the muscular side. Representative contact responses are shown in Fig. 8.

The ex vivo samples exhibited a similar signal reversal trend to that observed in the synthetic trachea models. However, greater variability in the FBG signal trend was observed, attributed to low mucosal friction which occasionally caused minor displacement of the sensor pusher.

Although the shapes of the contact sensor response curves differ across the cylindrical trachea models, bio-inspired trachea model, and ex vivo porcine samples, the onset of reversal in the FBG signal indicates the inflation pressure at which contact occurs.

**Fig. 9:**
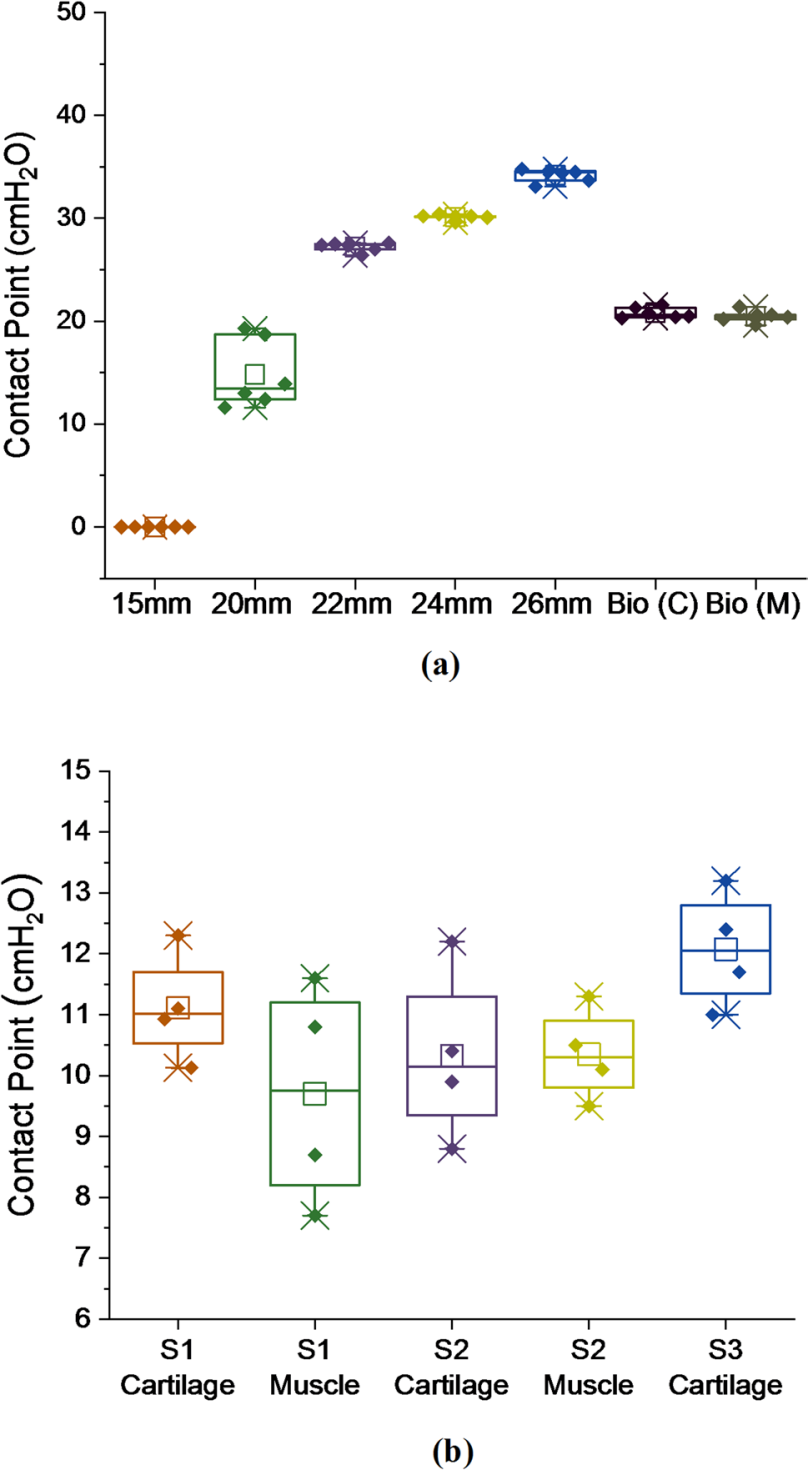
Box plot showing cuff inflation pressure when contact occurs with the wall of the tracheal model. **a** Cylindrical models (15 mm ID, 20 mm ID, 22 mm ID, 24 mm ID, 26 mm ID) and bio-inspired model (22 mm ID); cartilage (C) and muscle (M); **b** ex vivo porcine trachea (~ 15 mm ID, *n* = 3); cartilage and muscle. S3 muscle excluded due to sample damage. Horizontal line, median; box, interquartile range (IQR); whiskers, range; square, mean; diamond dots, data points

Six contact tests were performed for each trachea model (cylindrical and bio-inspired), with results summarised in Fig. 9a and individual repeats shown in Fig. S3. For the ex vivo porcine samples (*n* = 3), four contact tests were performed for each sample, with results summarised in Fig. 9b and individual repeats shown in Fig. S4.

For the five cylindrical trachea models, the pressure at which contact was established increased with increasing model internal diameter (15 mm ID: 0 ± 0 cmH_2_O; 20 mm ID: 14.8 ± 3.33 cmH_2_O; 22 mm ID: 27.2 ± 0.45 cmH_2_O; 24 mm ID: 30.1 ± 0.27 cmH_2_O; 26 mm ID: 34.2 ± 0.65 cmH_2_O). The muscle and cartilage results for the bio-inspired trachea model had the same mean contact point value (20.5 ± 0.59 cmH_2_O vs. 20.8 ± 0.53 cmH_2_O; *p* = 0.2808). Due to the small diameter, contact was observed from insertion of the tracheal tube for the 15 mm ID cylindrical model (0 ± 0 cmH_2_O). The 20 mm ID cylindrical model had the largest variability in contact test result repeats amongst the synthetic model contact tests (14.8 ± 3.33 cmH_2_O). The cuff contacted the 22 mm ID cylindrical model at a higher pressure than the 22 mm ID bio-inspired model (27.2 ± 0.45 cmH_2_O vs. 20.65 ± 0.79 cmH_2_O; *p* < 0.0001).

For the ex vivo trachea, samples 1 and 2 showed comparable inflation pressures at contact between cartilage and muscle regions, with no statistically significant difference (sample 1: 11.12 ± 0.9 cmH_2_O vs. 9.7 ± 1.81 cmH_2_O, *p* = 0.21; sample 2: 10.33 ± 1.42 cmH_2_O vs. 10.35 ± 0.75 cmH_2_O, *p* = 0.97). Cartilage-side contact for sample 3 (12.08 ± 0.94 cmH_2_O) fell within the range of the other samples; however, measurements from the muscle region were excluded due to tissue damage sustained during repeated testing. The initial successful measurement (*n* = 1, 11.4 cmH_2_O; Fig. 8c) confirmed that contact detection was achievable in that region.

### Tracheal model seal tests

**Fig. 10:**
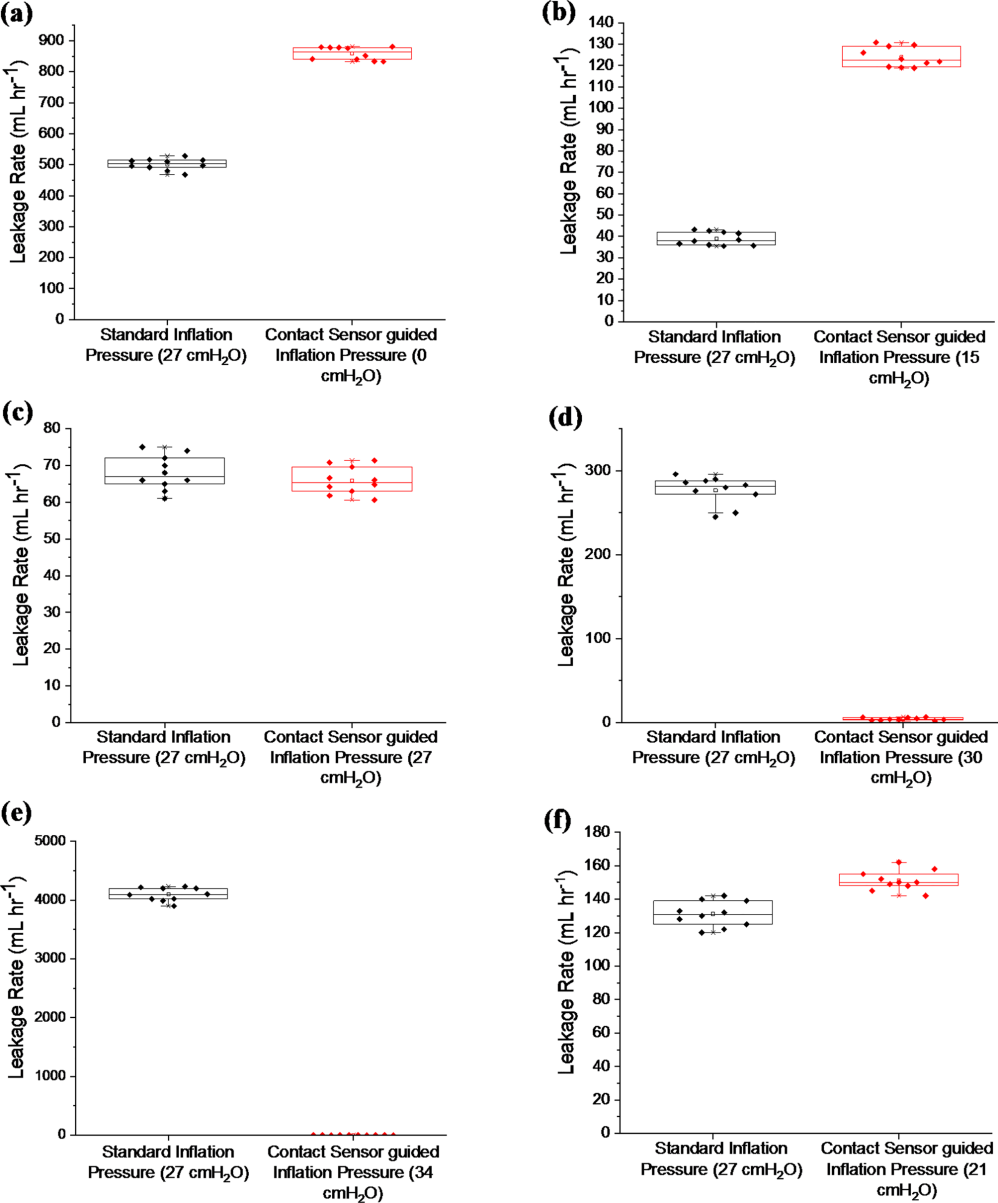
Box plot showing leakage rates for contact-sensing tracheal tube in the different cylindrical and bio-inspired trachea models. Horizontal line, median; box, IQR; whiskers, range; square, mean; diamond dots, data points. **a** 15 mm ID cylindrical model; **b** 20 mm ID cylindrical model; **c** 22 mm ID cylindrical model; **d** 24 mm ID cylindrical model; **e** 26 mm ID cylindrical model; **f** 22 mm ID bio-inspired model

Leakage rates were measured for the cylindrical and bio-inspired trachea models in two sets of experiments, with results shown in Fig. 10.

The seal test plot (Fig. 10) contains a comparison of leakage rates obtained at standard inflation pressure (27 cmH_2_O) with leakage rates obtained at the contact sensor guided inflation pressure for each model. A total of 20 tracheal seal test repeats were made for each model for the 2 experiments, 10 repeats per experiment. The mean leakage results are shown in Table S1.

Figure 10a includes the leakage rates for the 15 mm ID model. The contact-guided pressure was, 0 ± 0 cmH_2_O, as contact was already established from insertion of the tracheal tube, this resulted in an increase in mean leakage rate compared to the standard inflation pressure: 859 ± 21.03 mL hr^− 1^ vs. 501 ± 18.29 mL hr^− 1^; *p* < 0.0001. Leakage results for the 20 mm ID model are shown in Fig. 10b. Contact-guided pressure for the 20 mm ID model was 15 ± 3.33 cmH_2_O. This resulted in an increase in mean leakage rate compared to the standard inflation pressure: 124 ± 4.62 mL hr^− 1^ vs. 39 ± 3.09 mL hr^− 1^; *p* < 0.0001 following the trend of the 15 mm ID model. The 22 mm ID model leakage results are shown in Fig. 10c. The contact-guided pressure was 27 ± 0.45 cmH_2_O which is the same as the 27 cmH_2_O standard inflation pressure and the leakage rates were similar: 66 ± 3.74 mL hr^− 1^ vs. 68 ± 4.67 mL hr^− 1^; *p* = 0.3045. For the 24 mm ID cylindrical model, Fig. 10d, leakage at contact-guided pressure 30 ± 0.27 cmH_2_O resulted in a reduction in leakage compared to the standard inflation pressure: 4 ± 1.57 mL hr^− 1^ vs. 277 ± 16.85 mL hr^− 1^; *p* < 0.0001. The leakage result for the 26 mm ID cylindrical trachea model is shown in Fig. 10e. At 27 cmH_2_O, the mean leakage rate was 4096 ± 113 mL hr^− 1^ which was the highest amongst the five models. The contact-guided pressure was 34 ± 0.65 cmH_2_O which resulted in a reduction in leakage compared to the standard inflation pressure: 1 ± 0.53 mL hr^− 1^ vs. 4096 ± 113 mL hr^− 1^; *p* < 0.0001. The leakage tests in the cylindrical models showed lower leakage rates when the inflation pressure was set to the contact-guided pressure for the larger sized models; 24 mm ID and 26 mm ID. The 22 mm ID cylindrical model had a similar leakage rate for both tests, but the smaller sized cylindrical models; 15 mm ID and 20 mm ID had larger mean leakage rates at contact-guided pressure compared to the results at standard inflation pressure.

Leakage rates for the 22 mm ID bio-inspired model are shown in Fig. 10f. Leakage at contact-guided pressure (21 ± 0.75 cmH_2_O) resulted in a slight increase compared to the leakage at standard inflation pressure: 150 ± 5.25 mL hr^− 1^ vs. 131 ± 7.59 mL hr^− 1^; *p* < 0.0001.

## Discussion

An FBG-based contact sensor was developed for monitoring cuff–trachea interaction. FBG sensors were selected due to their immunity to electromagnetic interference, electrical isolation, compact size, and compatibility with integration within the cuff structure [24, 25]. In contrast, alternative sensing technologies such as piezoelectric, capacitive, or piezoresistive sensors, while sensitive, may present challenges related to humidity, electromagnetic interference, electrical safety, and integration within the confined cuff environment [26, 27]. Temperature cross-sensitivity of the FBG was mitigated using a cantilever configuration incorporating a second FBG. The mechanical durability of the embedded FBG sensor during repeated cuff inflation–deflation cycles is supported by the established use of fibre Bragg grating sensors in mechanically demanding environments, where they have demonstrated robustness under cyclic loading and long-term operation when appropriately protected [28, 29]. In the present design, the sensing region is encapsulated within a protective holder, and the remaining fibre is protected by a jacket. The system experiences minimal strain during cuff inflation–deflation, further reducing the likelihood of mechanical damage.

Contact was identified from the onset of reversal in the sensor response in all trachea models tested, with performance varying by diameter. The corresponding inflation pressure at contact was obtained from the synchronised manometer trace. The manometer measures intracuff pressure, whereas the FBG sensor records local deformation of the cuff wall. Non-uniform cuff expansion and the sensor’s placement on the cuff surface can produce transient variations in the sensor signal that are not reflected in the manometer pressure, although the contact point remains identifiable. Seal testing was conducted to compare standard inflation pressure with contact-guided inflation.

In smaller diameter cylindrical models (15 mm and 20 mm ID), increased variability in sensor response and higher leakage rates were observed, particularly in the 15 mm ID model, where contact occurred prior to inflation, indicating an ill-fitting cuff. This indicates that limited cuff expansion space results in cuff folding, reducing sealing performance [21]. In contrast, experimental measurements using larger diameter models (≥ 20 mm ID) showed more consistent behaviour, with improved cuff fit and reduced leakage when inflated to the contact-guided inflation pressure.

Experimental results with the bio-inspired model exhibited similar trends, with distinct differences in sensor response between the stiffer cartilage and the more compliant posterior muscle wall. Although similar mean contact-guided inflation pressures were observed for both regions, this may be explained by the behaviour of the high-volume low-pressure cuff, which expands to conform to the airway geometry and fills the asymmetric lumen during inflation, resulting in contact at comparable pressures. In addition, identification of contact is based on the onset of reversal in the FBG signal, with a minimum threshold of 0.01 nm applied to exceed the system noise level. This introduces a degree of insensitivity to minor geometric differences, as contact is only registered once sufficient displacement produces a measurable signal change.

When positioned over the muscle region, the sensor pusher sank into the softer lining, reflecting a limitation of the bio-inspired model, as the posterior tracheal wall is typically supported by the oesophagus in vivo. Nevertheless, this highlights the influence of local mechanical heterogeneity on sensor behaviour. While the contact-guided inflation pressures were similar, the sensor response profiles differed between regions: contact over cartilage (Fig. 7a) produced a sustained signal change, whereas contact over the compliant posterior wall (Fig. 7b) produced a shorter reversal followed by continued signal increase during inflation. Placing sensors on both cartilage and muscle regions may therefore assist in assessing cuff positioning and orientation during intubation. Although the bio-inspired model does not replicate all biological features of the human trachea, its contrasting cartilage and posterior wall stiffness provide a mechanically relevant and more physiologically representative platform than rigid cylindrical models for assessing cuff–trachea contact and contact-sensing feasibility. While the model represents a controlled benchtop approximation of the airway, the dominant mechanical heterogeneity of the trachea is preserved, enabling meaningful evaluation of cuff–trachea interaction under reproducible conditions.

Ex vivo testing using porcine trachea demonstrated that the contact-sensing tracheal tube could detect cuff–trachea contact in biologically derived airway tissue, with signal reversal observed in both cartilage and muscle regions. Greater signal variability was observed compared with the synthetic models, likely due to low mucosal friction affecting sensor stability and minor sensor displacement. However, the ex vivo evaluation was limited by the small sample size (*n* = 3) and variability inherent to biological tissue.

Porcine tracheas are widely used as experimental surrogates due to anatomical and mechanical similarities to human airways [22, 23], providing a physiologically relevant evaluation of device performance. Tissue damage during repeated testing highlights the limited durability of biological samples for mechanical measurements and reinforces the value of controlled synthetic models, such as the bio-inspired trachea used in this study, for repeatable device characterisation.

A consistent tracheal tube configuration was used to enable within-model comparison of standard and contact-guided inflation across varying airway geometries, reflecting standardised bench testing approaches (e.g. ISO 5361:2023). The tested tracheal diameters (15–26 mm) represent a range of airway geometries reported in the literature [30, 31]. In smaller diameter models, increased leakage and non-uniform cuff–wall contact were observed (Figs. 10a and b). This suggests that constrained cuff expansion results in cuff folding, contributing to reduced sealing efficiency. In larger models, more uniform cuff expansion and improved wall conformity were observed (Figs. 10d and e), corresponding to reduced leakage with contact-guided inflation. These behaviours are expected to be scalable across tube sizes, as similar trends were observed across the tested models. However, the use of a single tube size in this study may influence the observed leakage behaviour, as the degree of tube–airway mismatch affects cuff expansion and sealing performance. Therefore, the results should be interpreted in the context of the tested configuration. In clinical practice, cuff inflation is typically guided by intracuff pressure, which does not directly indicate cuff–trachea contact; the proposed sensor provides a means to access cuff–trachea interaction following tube placement.

A high-volume, low-pressure cuff was used, as this remains the most common choice in clinical practice [32, 33]. The development of HVLP cuffs has significantly improved tracheal intubation safety by reducing the risk of mucosal injury compared with traditional high-pressure cuffs, through more even pressure distribution over a larger surface area [32]. While HVLP cuffs reduce localised pressure points associated with ischaemia and tissue damage, they still rely on inflation pressure as a surrogate for cuff–trachea interaction.

Reliable monitoring of cuff-trachea interaction would enable more precise control of cuff inflation and may support renewed use of elastic materials in cuff design. Such materials could improve sealing performance by reducing cuff folding, particularly in smaller tracheal diameters, thereby lowering leakage and the associated risk of VAP [34].

The contact-sensing tracheal tube has been evaluated using bench tests, which are the most practical method for assessing cuff seal. This provides a basis for progression to in vivo use. Future miniaturisation of the sensor would facilitate integration within low-volume cuffs, minimise any influence on cuff mechanics, and broaden applicability across tracheal sizes.

## Conclusion

A sensor-equipped tracheal tube which informs cuff–trachea contact has been developed and evaluated using the ISO 5361:2023 tracheal seal bench testing method at both standard inflation pressure and contact-guided inflation pressure. The contact-sensing tracheal tube comprised two fibre Bragg grating-based optical fibre sensors attached to a cantilever and embedded within a high-volume low-pressure size 8 mm tracheal tube cuff. Contact and seal testing were performed across cylindrical trachea models of varying sizes and a bio-inspired trachea model, with contact testing further conducted in ex vivo porcine trachea samples.

Contact was observed in all five cylindrical trachea models (15 mm, 20 mm, 22 mm, 24 mm and 26 mm ID), the 22 mm ID bio-inspired model and was further demonstrated in ex vivo porcine trachea samples, confirming the feasibility of contact detection in biologically derived airway tissue. Seal test results for larger diameter models (24 mm and 26 mm ID) showed reduced leakage at contact-guided inflation pressure compared with standard inflation pressure, whereas the opposite trend was observed for smaller models (15 mm and 20 mm ID). No significant difference was observed for the 22 mm ID model, where inflation pressures at standard and contact conditions were comparable.

Cuff–trachea contact monitoring during intubation could provide clinical value by reducing the risk of cuff underinflation or overinflation. The proposed sensor has the potential to support personalised cuff inflation to accommodate variation in adult human tracheal size. In tracheal models sufficiently large to allow uniform cuff expansion, contact-guided inflation improved sealing performance. In smaller models, cuff folding introduced leakage pathways; although contact was detected, improved sealing was achieved using standard inflation pressure. These findings suggest that optimal performance may be achieved through a combination of contact sensing and a lower-volume cuff design. Further miniaturisation of the sensor would facilitate integration within low-volume cuffs, minimise any influence on cuff mechanics, and support future in vivo application.

## Supplementary Information

Below is the link to the electronic supplementary material.

Supplementary Material 1

## Data Availability

The data that support the findings of this study are available upon reasonable request from the corresponding author.
