# Supplementary material for: Bench testing a contact sensing tracheal tube for monitoring the cuff–trachea interface
Source: BMC Biomed Eng. 2026 Jun 1;8:10. doi: 10.1186/s42490-026-00113-y (PMC13224478; doi:10.1186/s42490-026-00113-y)
Supplement: Supplementary file 1 — Supplementary Material 1 [file 42490_2026_113_MOESM1_ESM.pdf]

Supplementary document for: Bench testing a contact sensing tracheal tube for monitoring the cuff-trachea interface.

FBG Cantilever Sensor

The FBG cantilever sensor tests were conducted using a digital manometer, 3-way stopcock and programmable syringe pump. The intracuff pressure was changed in steps of 5 cmH_2O.

The two FBGs bend in opposite directions during testing. Illustration of top and bottom FBG sensor response to applied pressure shown in Fig. S1.

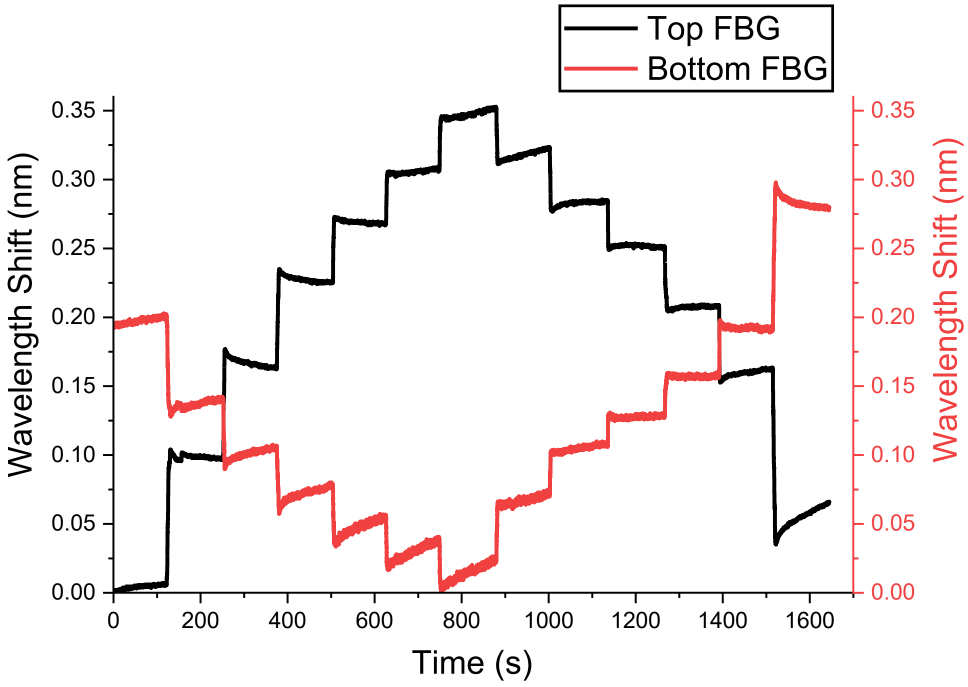

Fig. S1 Wavelength shifts from two FBG sensors during calibration testing, top FBG; black bottom FBG; red

The difference between the wavelength shifts compensates for temperature cross sensitivity. The differential wavelength shift was obtained from the two FBGs and plotted against the intracuff pressure as shown in Fig. S2.

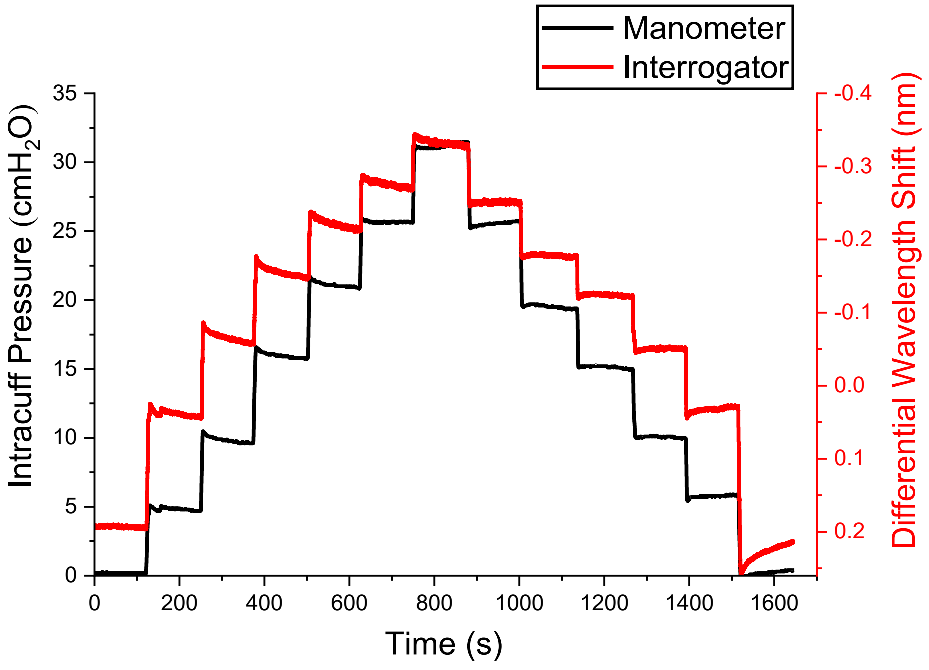

Fig. S2 FBG cantilever sensor response: differential wavelength shift vs intracuff pressure, intracuff pressure; black, differential wavelength shift; red

Contact Test Results for Cylindrical and Bio-inspired Models

Six contact tests were performed for each cylindrical and bio-inspired trachea model. Results for the 15 mm ID, 20 mm ID, 22 mm ID, 24 mm ID and 26 mm ID cylindrical models, and the 22 mm ID bio-inspired trachea model (cartilage and muscle), are included in Fig. S3.

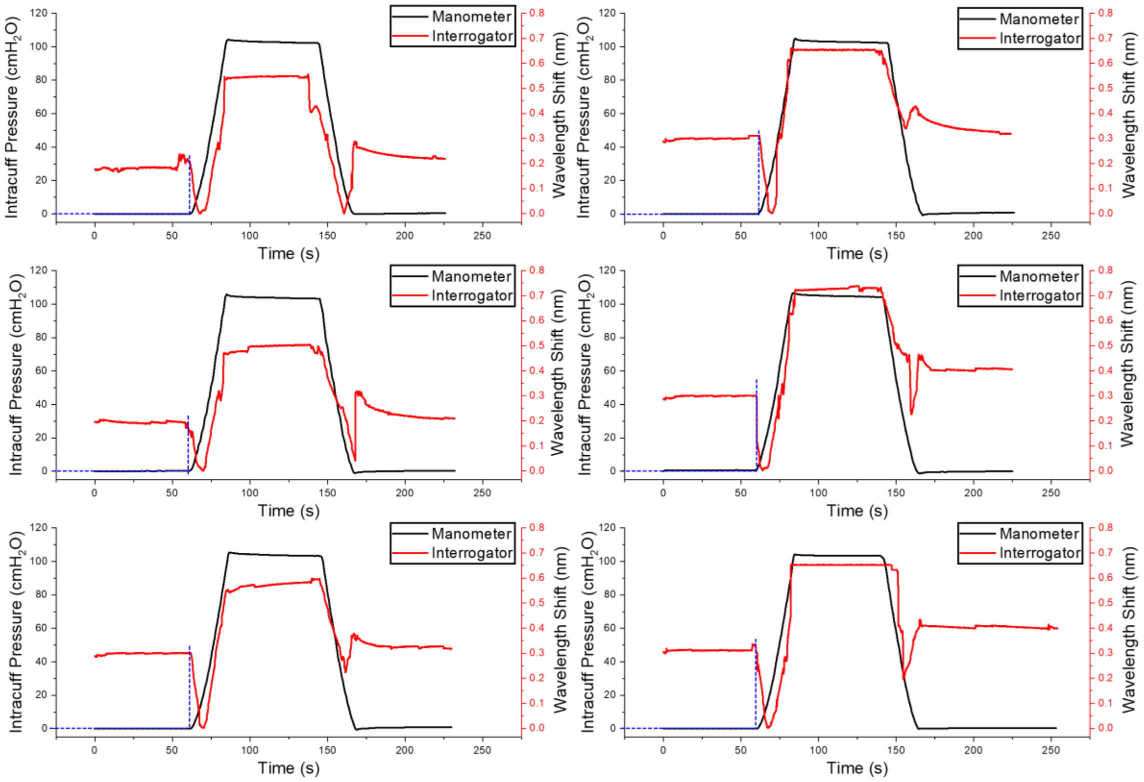
Manometer
Interrogator
Intracuff Pressure (cmH_2O)
Wavelength Shift (nm)
Time (s)

(a)

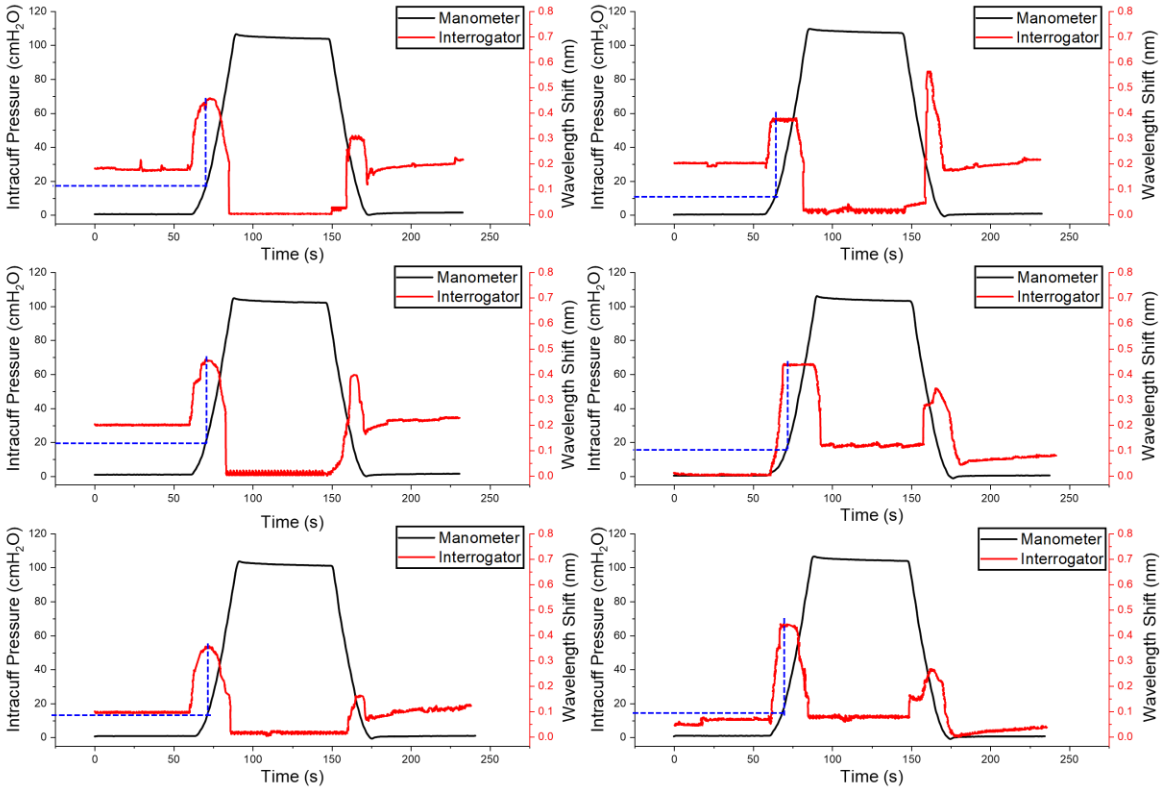
Manometer
Interrogator
Intracuff Pressure (cmH_2O)
Wavelength Shift (nm)
Time (s)

(b)

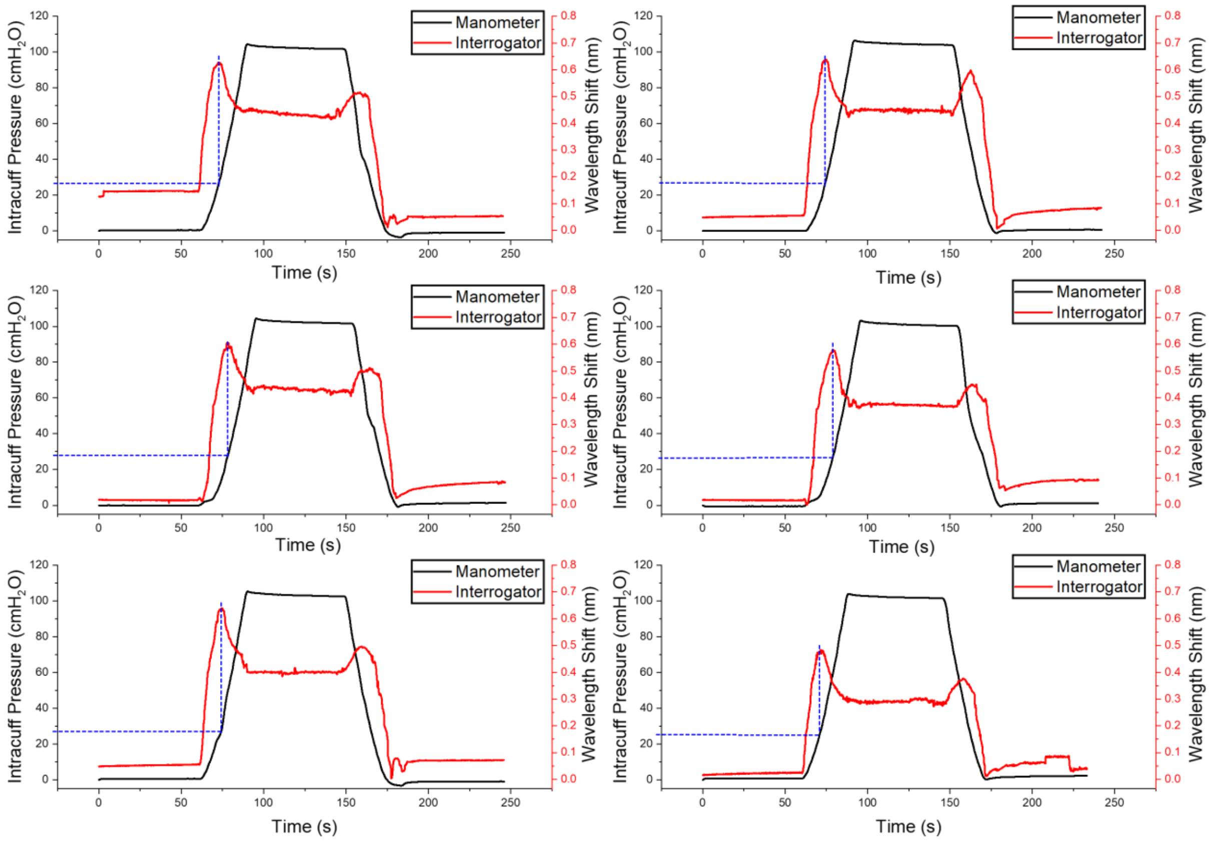
Manometer
Interrogator
Intracuff Pressure (cmH2O)
Wavelength Shift (nm)
Time (s)

(c)

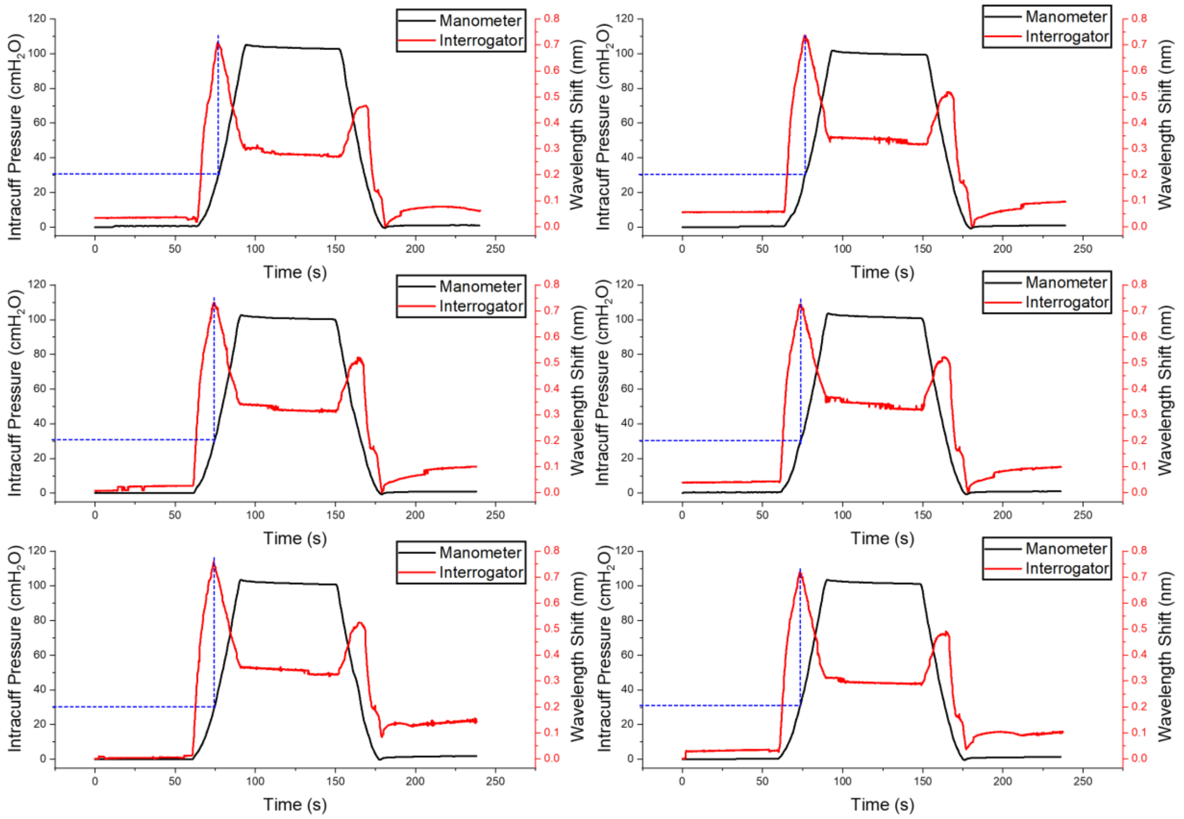
Manometer
Interrogator
Intracuff Pressure (cmH2O)
Wavelength Shift (nm)
Time (s)

(d)

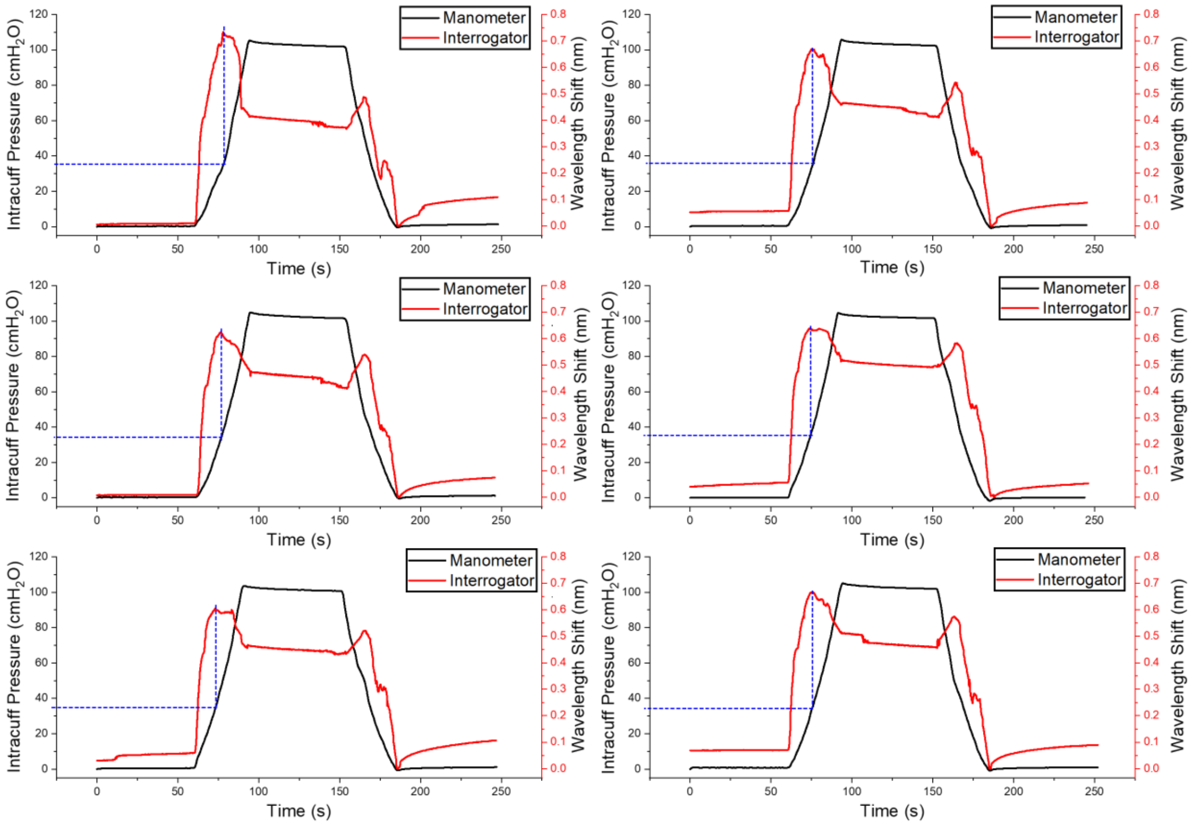
Manometer
Interrogator
Intracuff Pressure (cmH_2O)
Wavelength Shift (nm)
Time (s)

(e)

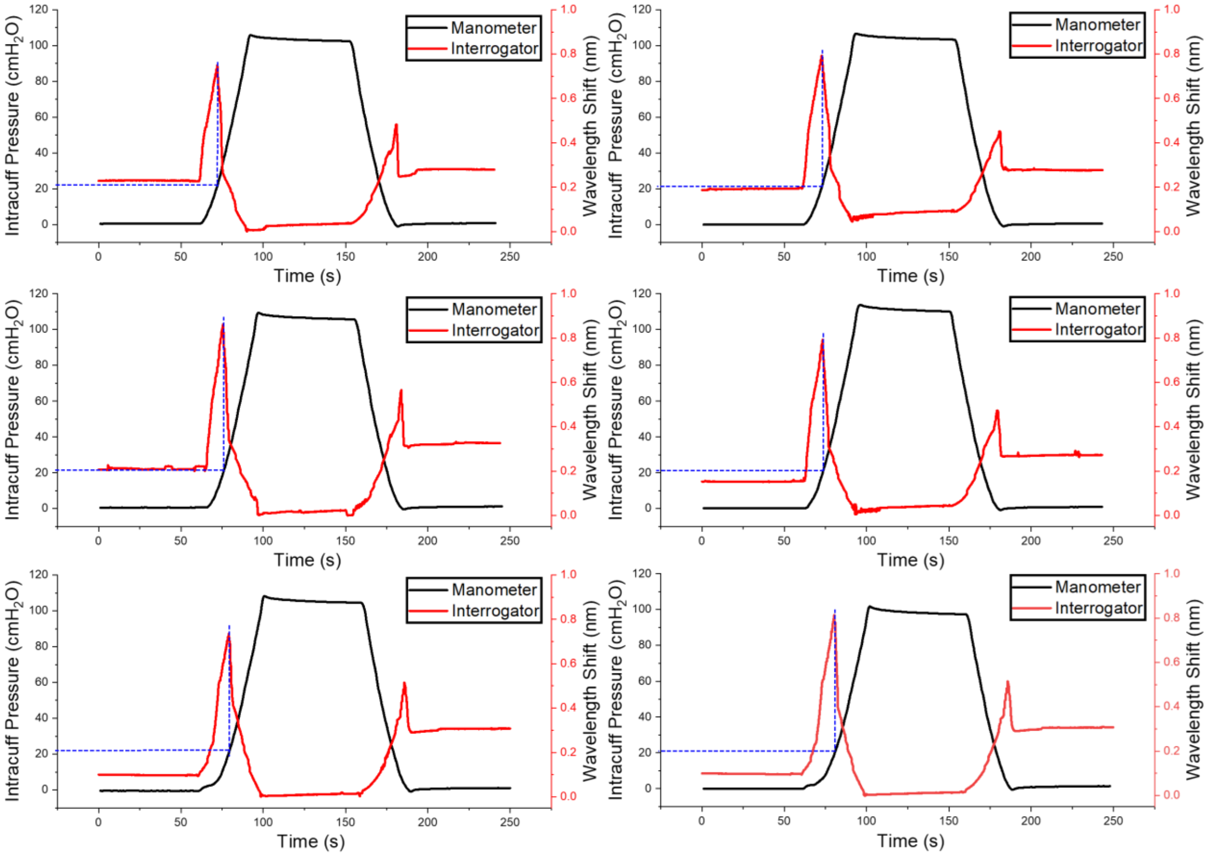
Manometer
Interrogator
Intracuff Pressure (cmH_2O)
Wavelength Shift (nm)
Time (s)

(f)

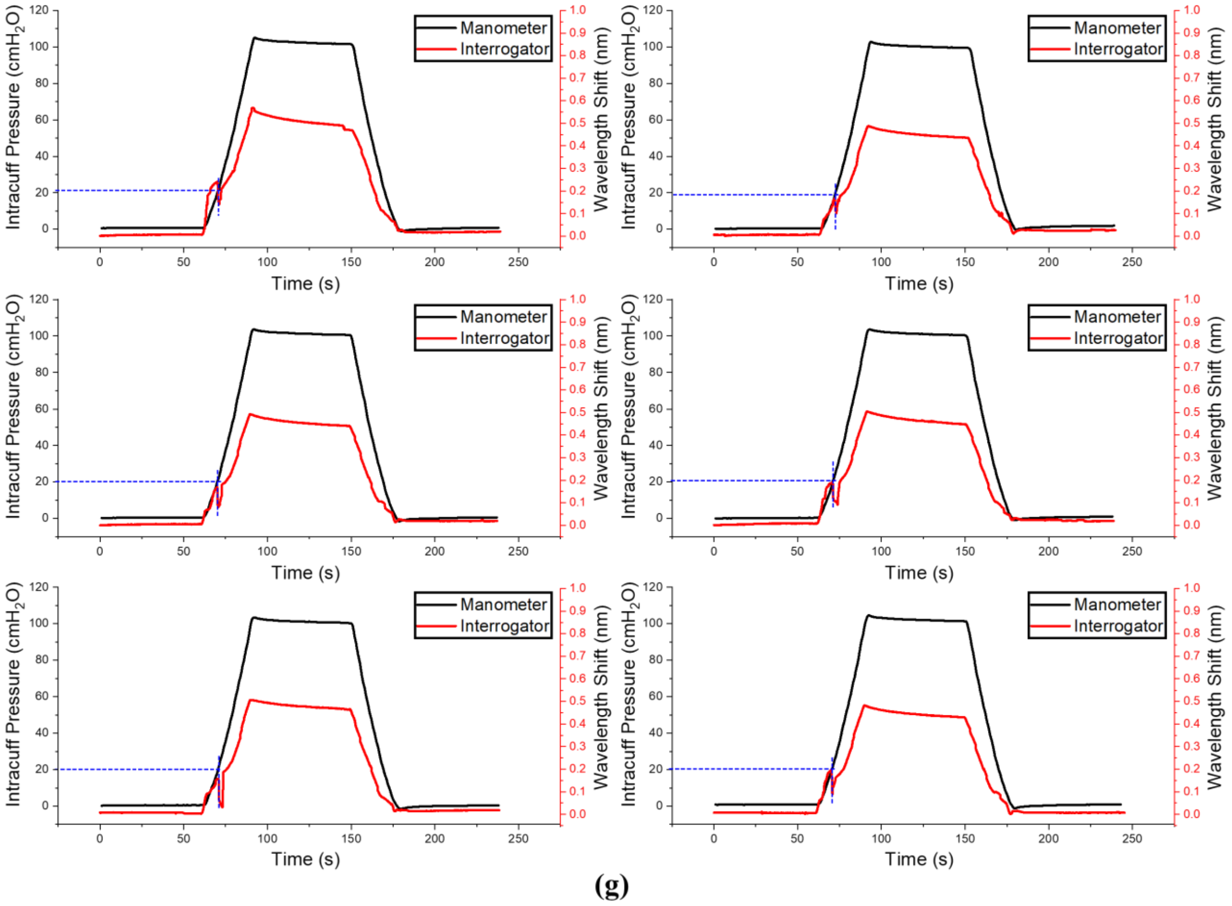

(g)

Fig. S3 Contact test results for trachea models. The blue dashed line indicates the inflation pressure at which contact occurs. **a** 15 mm ID cylindrical model; **b** 20 mm ID cylindrical model; **c** 22 mm ID cylindrical model; **d** 24 mm ID cylindrical model; **e** 26 mm ID cylindrical model; **f** 22 mm ID bio-inspired model: cartilage; **g** 22 mm ID bio-inspired model: muscle

Ex vivo Porcine Trachea Sample Contact Test Results

Four contact tests were performed for each *ex vivo* porcine trachea sample (15 mm ID), with results shown in Fig. S4.

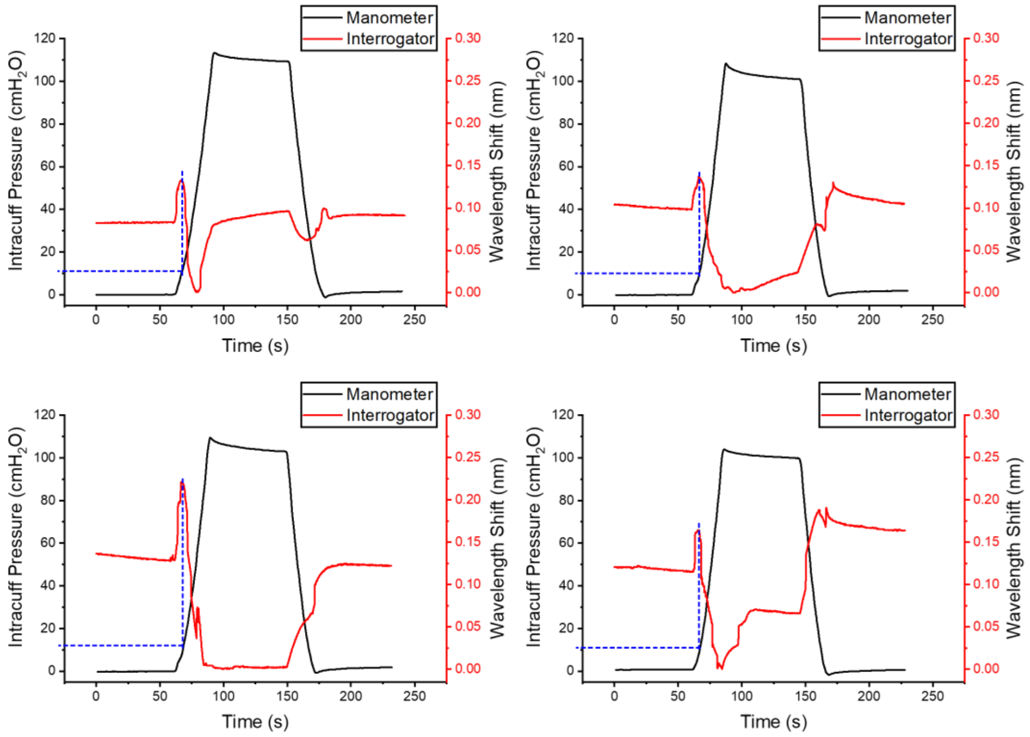
Manometer
Interrogator
Intracuff Pressure (cmH_2O)
Wavelength Shift (nm)
Time (s)

(a)

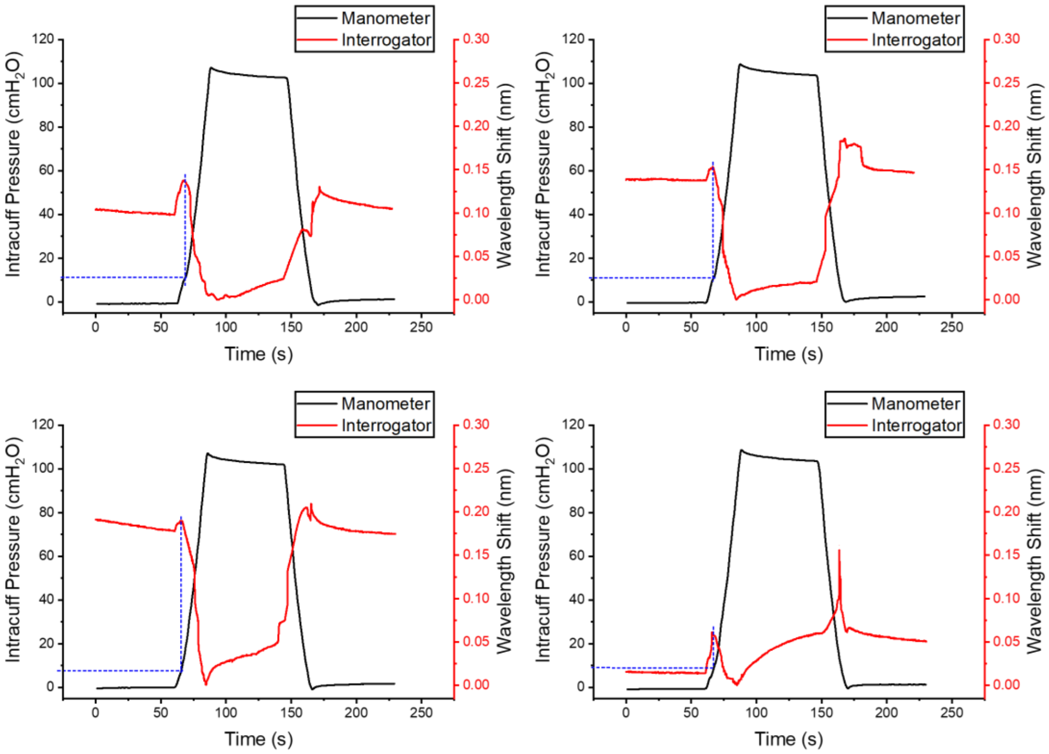
Manometer
Interrogator
Intracuff Pressure (cmH_2O)
Wavelength Shift (nm)
Time (s)

(b)

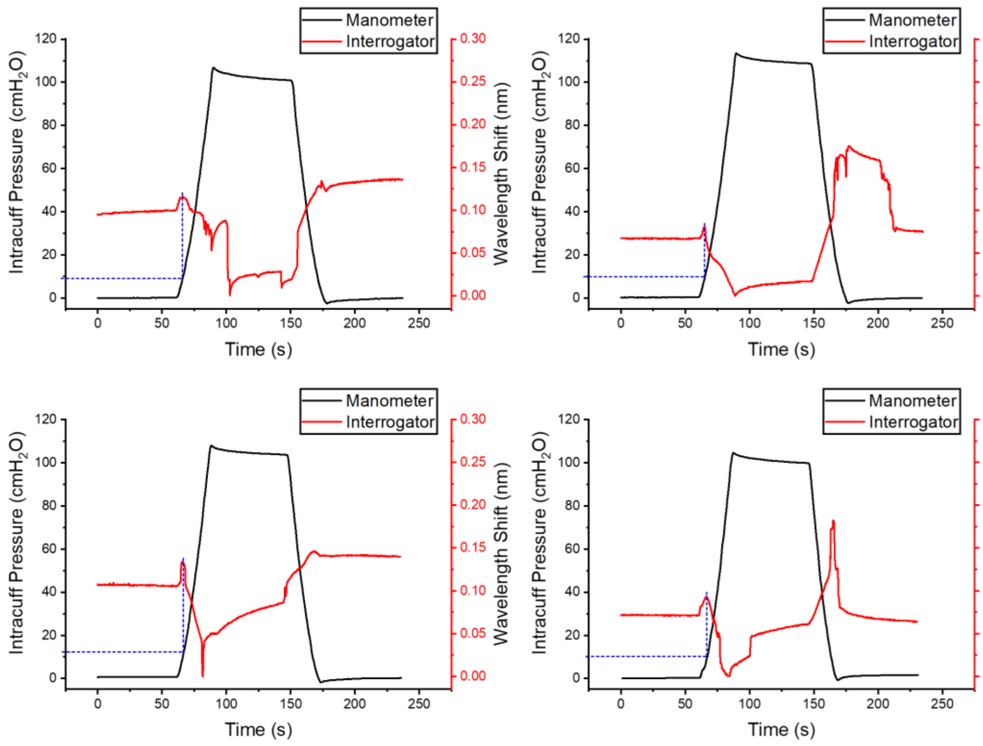
Manometer
Interrogator
Intracuff Pressure (cmH$_2$O)
Wavelength Shift (nm)
Time (s)
0
50
100
150
200
250
0
20
40
60
80
100
120
0.00
0.05
0.10
0.15
0.20
0.25
0.30
(c)

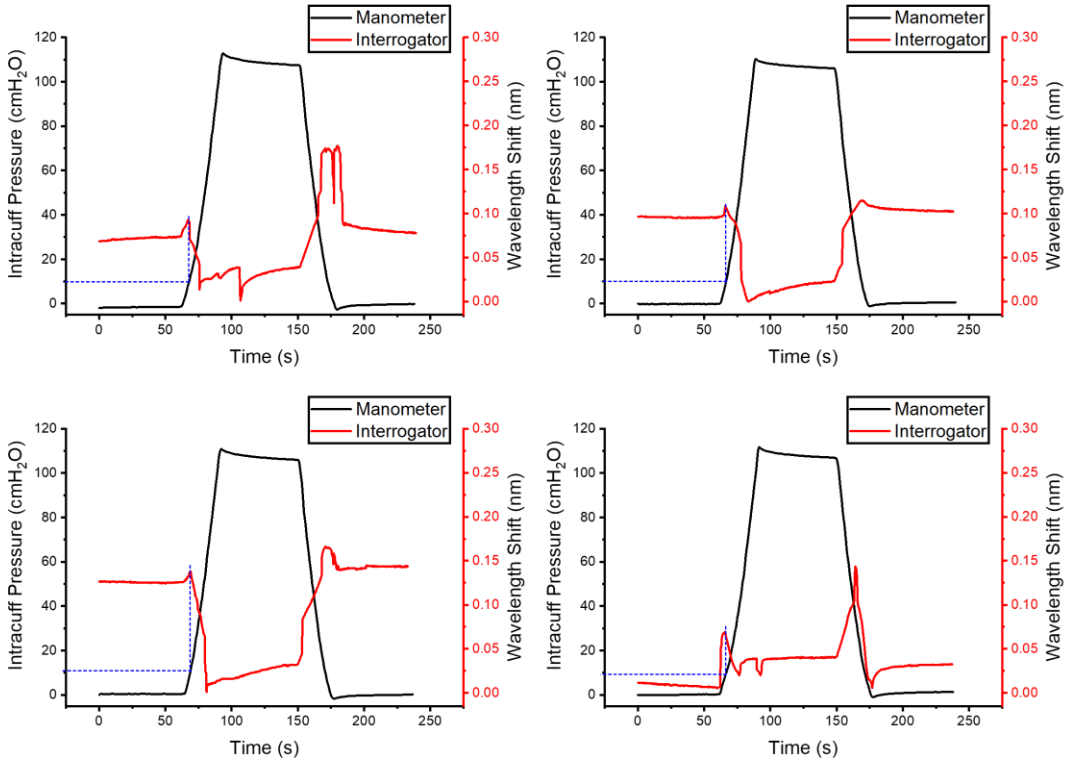
Manometer
Interrogator
Intracuff Pressure (cmH$_2$O)
Wavelength Shift (nm)
Time (s)
0
50
100
150
200
250
0
20
40
60
80
100
120
0.00
0.05
0.10
0.15
0.20
0.25
0.30
(d)

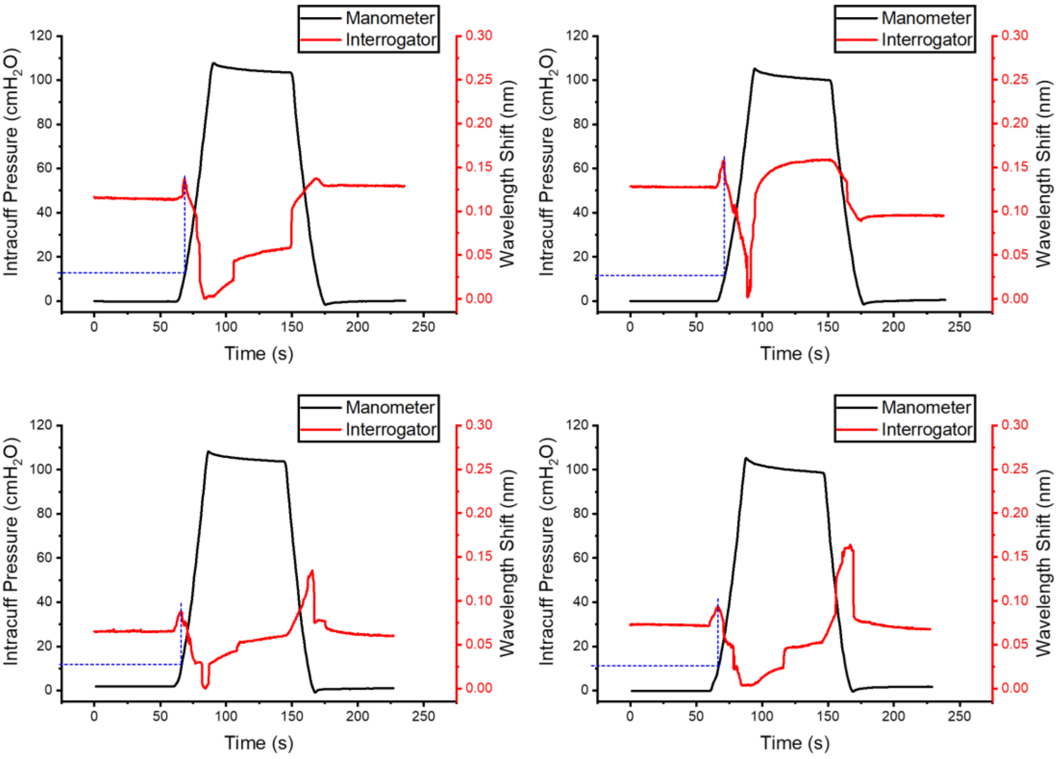

(e)

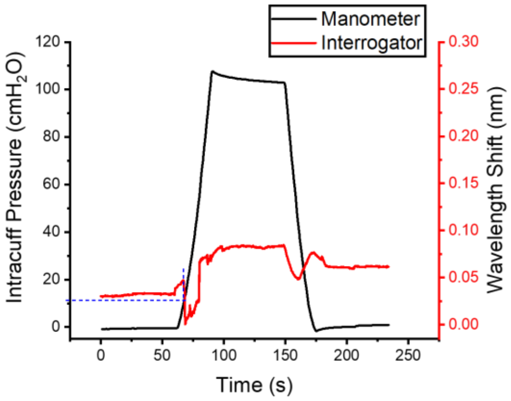

(f)

Fig. S4 Contact test results for 15 mm ID *ex vivo* porcine trachea samples. The blue dashed line indicates the inflation pressure at which contact occurs. **a** Sample 1 (Cartilage); **b** Sample 1 (Muscle); **c** Sample 2 (Cartilage); **d** Sample 2 (Muscle); **e** Sample 3 (Cartilage); **f** Sample 3 (Muscle; n = 1; Sample damaged after first test).

ISO Cuff Leakage Experiment

Procedure

EN ISO 5361:2023 recommends performing tests in 2 cylinders with diameters at the maximum and minimum of the range specified by the manufacturer. In this case we use 5 cylinders within the range for a size 8 cuff tracheal tube and a bio-inspired model. We test the contact-sensing tracheal tube and repeat measurements 20 times for each trachea model, 10 repeats at the recommended inflation pressure (27 cmH_2O) and 10 repeats at the contact sensor guided inflation pressure for the trachea model to estimate error bars in the results, the standard recommends testing 30 tracheal tubes once. Finally, the standard recommends conducting the whole experiment at 37°C, whereas although all components were warmed to 37°C beforehand, the experiment was conducted under ambient conditions.

Results

Leakage tests were conducted using the contact-sensing tracheal tube in 6 trachea models. Mean leakage rates shown in Table S1.

Table S1. Leakage rates for contact-sensing tracheal tube in the different cylindrical and bio-inspired trachea models at standard inflation pressure and contact sensor guided inflation pressure

**Trachea Model**	**Leakage Rate at Standard Inflation Pressure (mL hr^{-1}) [Std Dev]**	**Leakage Rate at Contact Sensor Guided Inflation Pressure (mL hr^{-1}) [Std Dev]**
15 mm ID Cylindrical Model	501 [18.29]	859 [21.03]
20 mm ID Cylindrical Model	39 [3.09]	124 [4.62]
22 mm ID Cylindrical Model	68 [4.67]	66 [3.74]
24 mm ID Cylindrical Model	277 [16.85]	4 [1.57]
26 mm ID Cylindrical Model	4096 [113.43]	1 [0.53]
22 mm ID Bio-inspired Model	131 [7.59]	150 [5.25]
